# Isocoumarin Synthesis via Metal-Free C-Arylation of Acetoacetates with *ortho*-Ester-Functionalized Diaryliodonium Salts

**DOI:** 10.3390/molecules31071069

**Published:** 2026-03-24

**Authors:** Elghareeb E. Elboray, Daichi Kashiwagi, Kotaro Kikushima, Mihoyo Fujitake, Toshifumi Dohi

**Affiliations:** 1 College of Pharmaceutical Sciences, Ritsumeikan University, 1-1-1 Nojihigashi, Kusatsu 525-8577, Shiga, Japan; 2 Department of Chemistry, Faculty of Science, South Valley University, Qena 83523, Egypt; 3 Center for Pharmaceutical Research and Development, Osaka Medical and Pharmaceutical University, 4-20-1 Nasahara, Takatsuki 569-1094, Osaka, Japan

**Keywords:** hypervalent iodine, ortho-functionalized iodonium salt, C-arylation, lactonization, isocoumarin

## Abstract

In this study, a metal-free approach was developed for the synthesis of isocoumarin frameworks by exploiting the reactivity between *ortho*-carboxylate-ester-substituted diaryliodonium salts and acetoacetates. This transformation involved the sequential C-arylation of an activated methylene substrate, followed by in situ enolization and intramolecular lactonization to construct an isocoumarin core. Under operationally simple conditions, a range of diaryliodonium salts and acetoacetate esters were employed to afford structurally diverse isocoumarins. The resulting products contained synthetically valuable functional groups, including halogen, nitro, carboxylate ester, and azide substituents, which facilitated further derivatization and extension toward complex architectures and potential applications. Subsequent transformation of the selected isocoumarin products enabled the synthesis of furo[3,4-c]isochromene-1,5-dione motifs, which are observed in several natural products.

## 1. Introduction

**Figure 1:**
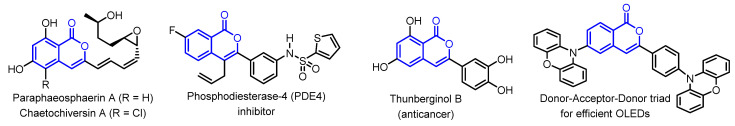
Examples of natural products and synthetically useful isocoumarin derivatives.

Isocoumarins (also referred to as 1*H*-isochromen-1-ones, 1*H*-2-benzopyran-1-ones, or 3,4-benzo-2-pyrones) are heterocyclic aromatic compounds consisting of a benzene ring fused to an unsaturated δ-lactone (α-pyranone) moiety. Isocoumarin derivatives represent an important class of naturally occurring metabolites found in various plants, molds, lichens, bacteria, and fungi. To date, nearly one thousand natural isocoumarin derivatives have been identified, displaying a diverse array of bioactivities, including cytotoxic, antibiotic, antifungal, antimalarial, antitubercular, antileukemic, antidiabetic, antifouling, antiplatelet, and antiviral effects [1,2,3,4,5,6,7,8]. Furthermore, isocoumarins serve as crucial intermediates in the synthesis of pharmaceutically active compounds [9,10,11], the construction of various isoquinolines, isochromenes, and aromatic derivatives [12,13,14,15], and the development of fluorescent materials for efficient organic light-emitting diode (OLED) devices (Figure 1) [16,17,18]. Consequently, the isocoumarin skeleton is regarded as a privileged structure with diverse applications in drug discovery, medicinal chemistry, materials science, and organic synthesis [19,20,21,22].

Owing to their diverse biological activities and significance as key scaffolds in synthetic chemistry, considerable research efforts have been dedicated toward the synthesis of isocoumarin derivatives. Traditional synthetic methods typically involve multistep processes and harsh reaction conditions, resulting in low yields and regioselectivities, along with the formation of undesired byproducts [12,13,14,15]. For instance, the reaction of *ortho*-halobenzoic acids with 1,3-diketones leads to additional diacylation, yielding 4-unsubstituted isocoumarin derivatives under both metal- and metal-free conditions [23,24,25,26]. Consequently, advanced methodologies have been developed using transition metal catalysts, such as palladium, copper, ruthenium, rhodium, and iridium [27,28,29,30,31,32]. Common synthetic strategies involve the formation of *ortho*-alkynylbenzoic acids or their ester/amide derivatives, followed by intramolecular cyclization via one-pot cascades or multistep processes. Besides transition metal-catalyzed conditions, Lewis/Brønsted acids and electrophilic reagents have been employed to facilitate these cyclization reactions [27,28,29,30,31,32]. Metal-catalyzed processes have also been used to promote the annulation of benzoic acid derivatives with ketones, alkynes, alkenes, diazoalkanes, and sulfonium ylides, among other substrates.

**Scheme 1:**
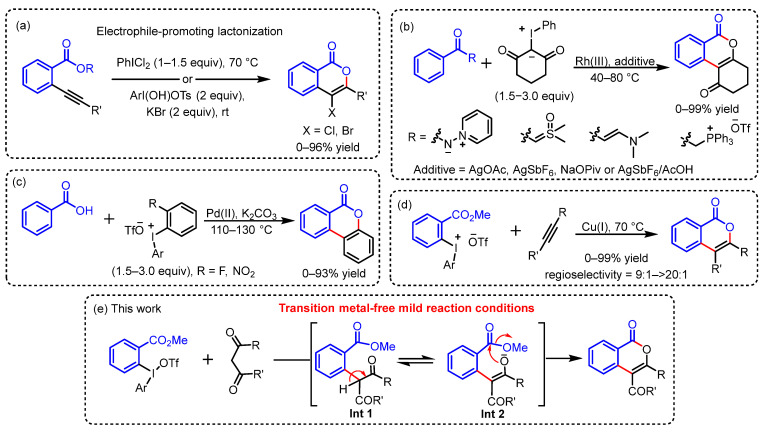
Participation of hypervalent iodine species in the synthesis of isocoumarin derivatives through (**a**) intramolecular cyclization of *ortho*-alkynylbenzoate; (**b**) Rh-catalyzed annulation with aroyl derivatives; (**c**) Pd-catalyzed cyclization with aryl carboxylic acid; (**d**) Cu-catalyzed annulation with alkyne; and (**e**) metal-free with β-ketoester.

Hypervalent iodine reagents and diaryliodonium salts are either commercially available or readily prepared, and they exhibit broad applicability [33,34]. For example, (dichloroiodo)benzene has been employed as both an oxidant and chlorinating agent for the intramolecular cyclization of *ortho*-alkynylbenzoate, leading to the formation of 4-chloroisocoumains (Scheme 1a) [35]. Additionally, a combination of the Koser reagent and potassium bromide has been used as an electrophilic reagent system for the brominative annulation of *ortho*-alkynylbenzoate, enabling the synthesis of 4-bromoisocoumarin derivatives [36]. Furthermore, hypervalent iodonium ylides have been annulated with iminopyridinium ylides, sulfoxonium ylides, phenacyl phosphoniums, and *N*,*N*-dimethyl enaminones via rhodium(III)-catalyzed C–H activation, carbene insertion, and nucleophilic addition protocols (Scheme 1b) [37,38,39,40,41]. Unsymmetrical scaffolds afforded mixtures of regioisomers, whereas iodonium ylides bearing 5-/7-membered cyclic 1,3-diones gave low yields, and those derived from acyclic 1,3-diones were unreactive. Moreover, *ortho*-functionalized aryliodonium salts effectively participated in the construction of benzopyranone skeletons under transition metal-catalyzed conditions. Iodonium salts substituted with *ortho*-fluoro and -nitro groups were efficiently cyclized with aryl carboxylic acids under palladium(II) acetate conditions to produce the corresponding pyranone derivatives (Scheme 1c) [42,43]. In these reactions, palladium-promoted nucleophilic substitution of the *ortho*-nitro and *ortho*-fluoro groups by a coordinated carboxylate facilitates intramolecular lactonization. Generally, polynuclear aryl carboxylic acids cyclize more effectively than simple benzoic acid derivatives. In this context, *ortho*-carboxylate-ester-functionalized aryliodonium salts were successfully annulated with naphthols in the presence of a Cu catalyst to afford isocoumarin derivatives; however, the yields decreased when phenols were used, resulting in the formation of regioisomers [44]. Notably, aryliodonium salts substituted with *ortho*-carboxylate esters underwent annulation with alkynes under Cu catalysis to produce a diverse range of substituted isocoumarins in high yields with excellent regioselectivity (Scheme 1d) [45].

Previously, our group pioneered metal-free cross-coupling reactions employing diaryliodonium salts for C–C bond formation, including the synthesis of heteroaromatic biaryl compounds [46,47]. Our group further demonstrated the versatility of these C-, O-, and N-arylation protocols and established a practical, transition-metal-free approach for the synthesis of benzisoxazolone heterocycles from *ortho*-carboxylate-ester-substituted aryliodonium salts and protected hydroxylamines [48,49,50,51,52]. Building on this longstanding interest, the aim of the current study is to develop a mild, metal-free approach to isocoumarin derivatives via the C-arylation of activated methylenes with *ortho*-carboxylate-ester-substituted diaryliodonium salts, followed by intramolecular lactonization in a single step (Scheme 1e).

## 2. Results and Discussion

Diaryliodonium salts are stable, low-toxicity arylating agents that are extensively used in the construction of diverse C–C and C–heteroatom bonds under both metal-free and metal-catalyzed conditions [34,53,54]. Notably, metal-free transformations have garnered considerable interest as efficient and sustainable synthetic methodologies [34]. Additionally, the advanced design of diaryliodonium salts has facilitated the highly selective arylation of a wide array of nucleophiles, enhancing the practicality of these reactions and addressing the limitations associated with classical iodonium salts [46,47,55,56,57,58].

**Scheme 2:**
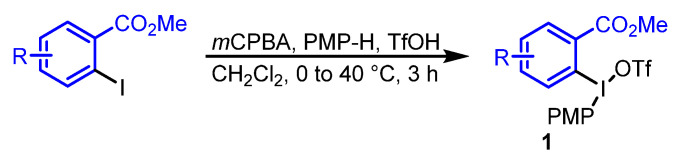
Synthesis of *ortho*-carboxylate-ester-functionalized diaryliodonium salts **1**.

*ortho*-Carboxylate-ester-functionalized diaryliodonium triflates **1** are easily accessible substrates through oxidation of commercially available *ortho*-carboxylate-ester-substituted (hetero)aryl iodides with *meta*-chloroperbenzoic acid (*m*CPBA), then coupling with anisole (PMP-H) in the presence of trifluoromethanesulfonic acid (TfOH) (Scheme 2) [59]. Aryl iodide scaffolds substituted with electron-rich, electron-poor, and sterically hindered groups were transformed smoothly into the related *ortho*-carboxylate-ester-functionalized Ar(PMP)IOTf **1**.

As a continuation of our investigations into *ortho*-carboxylate-ester-functionalized diaryliodonium salts bearing a *para*-methoxyphenyl (PMP) dummy ligand to synthesize valuable heterocyclic products [59], a new approach was developed for the synthesis of isocoumarin derivatives. The feasibility of this transformation was initially assessed using *ortho*-carboxylate-ester-substituted aryl(PMP)iodonium triflate **1a** and methyl acetoacetate **2a**, and subsequent optimization of the reaction parameters. Preliminary screening of commonly used conditions for the C-arylation of activated methylene scaffolds [60,61], including sodium hydride and potassium *tert*-butoxide in *N*,*N*-dimethylformamide (DMF), resulted in trace amounts of the desired product (Table 1, entries 1 and 2; Table S1). Notably, our previously reported reaction conditions afforded isocoumarin **3aa** in 66% yield (entry 3). Reducing the loading of iodonium salt **1a** to 1.0 or 1.5 equivalents resulted in decreased yields (54 and 60%, respectively), whereas increasing the amounts of methyl acetoacetate **2a** and base to 1.5 and 3.0 equivalents yielded isocoumarin **3aa** in 50 and 62% yields, respectively (Table S1). Conducting the reaction in dry diethyl ether without 4 Å molecular sieves gave a comparable yield (entry 4). Reduced solvent volumes (i.e., higher reactant concentrations) were detrimental to product formation (entries 5 and 6). Variations in the reaction temperature and time for the reaction employing iodonium salt **1a** (1–2 equiv.) failed to increase the yield of product **3aa** (entries 7 and 8; Table S1). Subsequent screening of organic and inorganic bases, along with a range of nonpolar and polar (a)protic solvents, identified cesium carbonate and diethyl ether as the optimal base and solvent, respectively (entries 9–16; Table S1). Remarkably, commercially available diethyl ether used as received afforded isocoumarin **3aa** in 77% isolated yield, whereas a mixed solvent system (diethyl ether/water) led to the complete consumption of iodonium salt **1a**, but resulted in a poor yield or no detectable formation of the desired product (entries 17 and 18; Table S1). In contrast, other *ortho*-carboxylate-substituted diaryliodonium salts bearing mesityl (Mes), 2,4,6-trimethoxyphenyl (TMP), or 3,5-dimethyl-4-isoxazolyl (DMIX) dummy aryl groups in combination with tosylate or triflate counteranions, afforded the corresponding products in lower yields (Table S2).

**Scheme 3:**
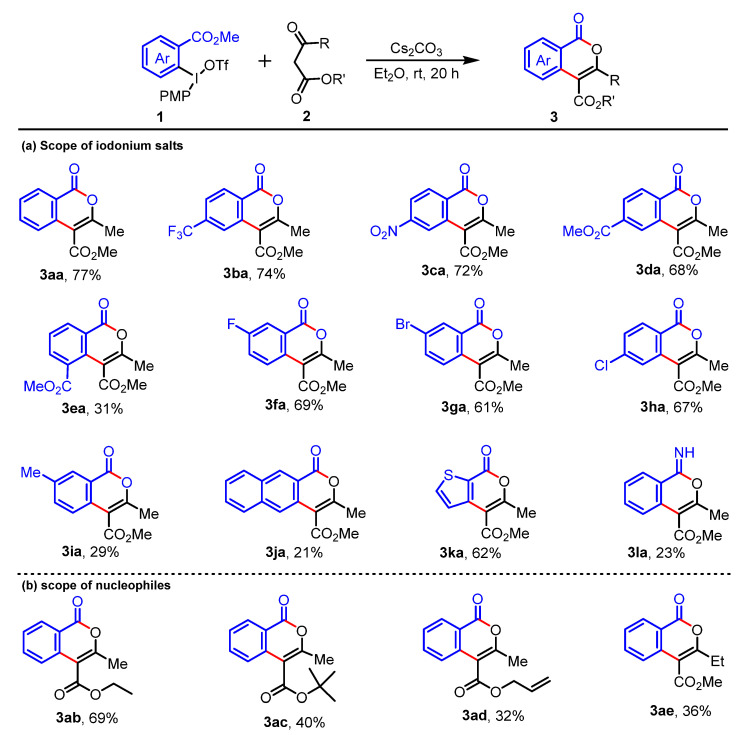
Scope of the iodonium salt in the reaction with acetoacetates to afford a range of isocoumarins.

With the optimized conditions in hand (Table 1, entry 17), the substrate scope of *ortho*-carboxylate-ester-substituted aryl(PMP)iodonium triflates was investigated in their reaction with methyl acetoacetate **2a** to access a range of isocoumarin derivatives (Scheme 3). Iodonium salts **1b**–**1d** substituted with various electron-withdrawing groups—including CF_3_, NO_2_, and CO_2_Me—reacted efficiently, affording the corresponding isocoumarin derivatives **3ba**–**3da** in high yields. Sterically hindered iodonium salt **1e**, characterized by its 2,6-dicarboxylate ester substitution pattern, reacted with acetoacetate **2a** to produce novel isocoumarin **3ea**, which contains 4,5-dicarboxylate ester groups, in moderate yield. Generally, sterically hindered substrates pose challenges to isocoumarin synthesis under both metal-catalyzed and metal-free conditions [35,36,37,38,39,40,43,45]. Consequently, novel isocoumarin **3ea** may be of particular interest and represents a potentially useful analog of widely employed naphthalene-1,8-dicarboxylate esters [62]. Additionally, iodonium salts **1f**–**1h** functionalized with halogen substituents (F, Br ad Cl) afforded the corresponding isocoumarins **3fa**–**3ha** in 69–61% yields, respectively. These halogenated products, which are often difficult to obtain under metal-catalyzed conditions, offer valuable handles for further derivatization. Furthermore, methyl-substituted iodonium salt **1i** and naphthyliodonium salt **1j** produced the corresponding isocoumarins **3ia** and **3ja** in moderate yields. Notably, heterocyclic iodonium salt **1k** reacted efficiently to furnish the thienopyranone skeleton **3ka** in good yield. *ortho*-Cyano-functionalized iodonium salt **1l** was also compatible with this approach, affording imino derivative **3la** in 23% yield.

Subsequently, the substrate scope was expanded by employing acetoacetates bearing various ester groups. The reactions of acetoacetates **2b** and **2c** furnished the corresponding isocoumarin products **3ab** and **3ac**, with yields reflecting the steric demands of the ester substituents. Notably, an increase in ester bulk from ethyl to *t*-butyl resulted in a pronounced decrease in yield from 69 to 40%. Additionally, allyl acetoacetate **2d** produced allyl ester isocoumarin **3ad** in 32% yield. Furthermore, the reaction of methyl 3-oxopentanoate **2e** with iodonium salt **1a** afforded the corresponding isocoumarin **3ae** in moderate yield. Thus, at this stage of the investigation, the method’s applicability appeared limited to a single class of activated methylene compounds. Similar substrate-dependent limitations have frequently been reported in C-arylation reactions of activated methylenes with diaryliodonium salts [61,63,64]. Indeed, these systems are susceptible to side reactions, including aldol addition/condensation of β-ketoesters [65], deacylation of C-arylation products [23,24,25,26,60,66], double arylation of activated methylenes [60,67,68], and decomposition of iodonium salts [69,70,71]. Within this context, the current protocol therefore represents a reliable metal-free approach that enables the formation of two bonds in a single operation, affording isocoumarin derivatives via C-arylation followed by intramolecular lactonization.

**Scheme 4:**
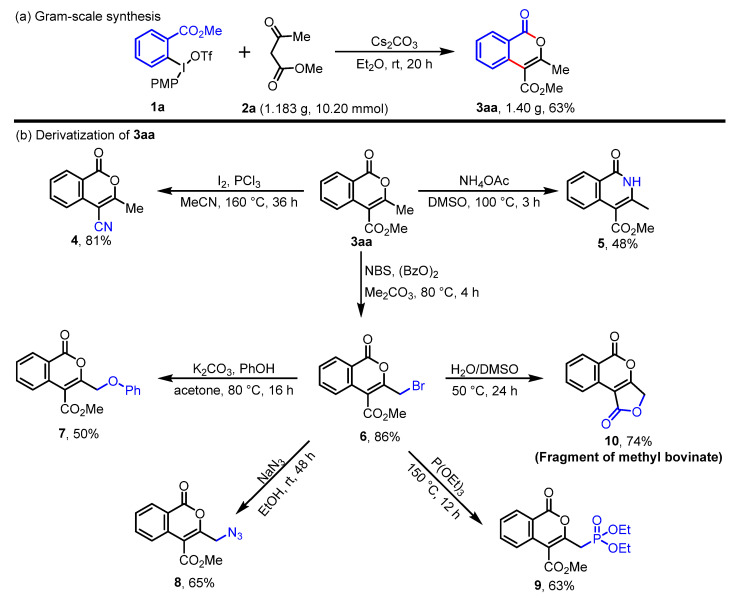
Gram-scale synthesis (**a**) and derivatization (**b**) of isocoumarin **3aa**.

These conditions were also applicable to large-scale synthesis, affording the desired isocoumarin product **3aa** in 63% yield (Scheme 4a). To illustrate the synthetic value of the isocoumarin derivatives, **3aa** was converted into valuable intermediates. Specifically, the 4-ester group of isocoumarin **3aa** was efficiently transformed into 4-cyano-derivative **4** via reaction with acetonitrile in the presence of I_2_/PCl_3_ (Scheme 4b) [72]. Additionally, treatment of **3aa** with ammonium acetate afforded isoquinoline derivative **5** in moderate yield, while the reaction of **3aa** with *N*-bromosuccinimide (NBS) afforded 3-bromomethyl derivative **6** in 86% yield. This derivative serves as a useful precursor for further functionalization; nucleophilic substitution of the bromo-group in **6** with phenol, azide, and triethyl phosphite afforded the corresponding ether-, azide-, and phosphonate derivatives **7**–**9** in good yields. Notably, azide-functionalized isocoumarin **8** has extensive applications in click chemistry. Moreover, the substitution of bromo-derivative **6** with water generated the corresponding alcohol, which underwent lactonization with the adjacent carboxylate ester group to form furo[3,4-c]isochromene-1,5-dione **10** in 74% yield. This furoisochromene–dione skeleton is the main fragment of the natural product methyl bovinate [73]. Notably, compounds analogous to **10** have previously been reported via a Rh(III)-catalyzed regioselective annulation of benzoic acid with 4-hydroxy-2-alkynoates, followed by intramolecular lactonization [74]. That method afforded a mixture of regioisomeric products and required a transition metal catalyst in combination with AgSbF_6_ as an activator, Ag_2_CO_3_ as an oxidant, and LiOAc as a base, and was performed at 100 °C for 16 h. In contrast, the present protocol delivers compound **10** as a single regioisomer in significantly higher yield under metal-free conditions, without expensive silver additives or external oxidants, and proceeds under milder and operationally simpler conditions.

## 3. Materials and Methods

### 3.1. General Information

Melting points were measured using a Yanaco MP-500P melting point (Yanagimoto Scientific Instruments, Kyoto, Japan) apparatus and are uncorrected. Nuclear magnetic resonance (NMR) spectra were recorded on a JEOL JMN-AL400 spectrometer (JEOL Ltd., Tokyo, Japan, ^1^H NMR: 400 MHz; ^13^C NMR: 101 MHz) at 25 °C. Chemical shifts in the ^1^H NMR spectra were referenced to the residual solvent signal (CDCl_3_, δ 7.26), and chemical shifts in the ^13^C NMR spectra were referenced to the residual solvent signal (CDCl_3_, δ 77.0). Data are reported as follows: chemical shift in ppm (δ), multiplicity (s = singlet, d = doublet, t = triplet, m = multiplet), coupling constant (Hz), and integration. Infrared (IR) spectra were obtained using a JASCO FT/IR-4200 (JASCO, Tokyo, Japan) spectrometer; absorptions are reported in reciprocal centimetres (cm^−1^) for strong and structurally diagnostic peaks. High-resolution mass spectra (HRMS) were obtained using the electron ionization (EI) method and recorded using a JMS-700 spectrometer (JEOL Ltd., Tokyo, Japan). Flash column chromatography and analytical thin-layer chromatography (TLC) were performed on Merck silica gel 60 (230–400 mesh) and Merck silica gel F_254_ plates (0.25 mm), respectively. Spots and bands were visualized by UV irradiation (254 nm). All reactions requiring heating were carried out using an IKA^®^ C-MAG HS 4 digital hotplate (IKA-Werke GmbH & Co. KG, Staufen im Breisgau, Germany) equipped with aluminium heating blocks. Room temperature in this work refers to a range of 25–30 °C. All commercially available reagents were used as received unless otherwise noted.

### 3.2. General Procedure for the Synthesis of Iodonium Triflates ***1*** [59]

To a stirred solution of aryl iodide (1.0 mmol) in DCM (5 mL, 0.2 M) were added *m*-CPBA (1.28 equiv) and anisole (1.0 equiv), and the reaction mixture was cooled to 0 °C. TfOH (2.0 equiv) was then added dropwise, and the mixture was stirred at 0 °C for 10 min and subsequently at 40 °C for 3 h. The solvent was removed under reduced pressure, and the resulting residue was triturated with Et_2_O to afford the desired aryl(PMP)iodonium triflate.

*ortho*-Ester-substituted aryl(PMP)iodonium triflates **1a**–**1d** and **1g**–**1k** and their corresponding cyano-substituted analogues were prepared according to our previous work [59].

Dimethyl 2-((4-methoxyphenyl)(((trifluoromethyl)sulfonyl)oxy)-λ^3^-iodaneyl)isopht-halate (**1e**). Gray amorphous solid (295.1 mg, 51%). M.p. 148–149 °C. ^1^H NMR (400 MHz, CDCl_3_ + drop CD_3_OD): *δ* 8.14 (d, *J* = 7.8 Hz, 2H, Ar–H), 7.83 (d, *J* = 8.8 Hz, 2H, Ar–H), 7.72 (t, *J* = 7.6 Hz, 1H, Ar–H), 6.88 (d, *J* = 8.8 Hz, 2H, Ar–H), 3.85 (s, 6H, 2 × CO_2_Me), 3.78 (s, 3H, OMe). ^13^C{1H} NMR (101 MHz, CDCl_3_ + drop CD_3_OD): *δ* 165.7, 163.0, 138.5, 135.7, 133.9, 132.5, 117.2, 115.5, 103.5, 55.8, 54.1.

Methyl 5-fluoro-2-((4-methoxyphenyl)(((trifluoromethyl)sulfonyl)oxy)-λ^3^-iodaney-l)benzoate (**1f**). Gray amorphous solid (285.9 mg, 64%). M.p. 120–121 °C. ^1^H NMR (400 MHz, CDCl_3_): *δ* 8.01–7.98 (m, 1H, Ar–H), 8.01 (d, *J* = 8.8 Hz, 2H, Ar-H), 7.34–7.30 (m, 1H, Ar–H), 7.10 (d, *J* = 8.8 Hz, 2H, Ar–H), 6.96 (dd, *J* = 9.0, 4.6 Hz, 1H, Ar–H), 4.12 (s, 3H, CO_2_Me), 3.91 (s, 3H, OMe). ^13^C{^1^H} NMR (CDCl_3_): *δ* 167.0 (d, *J* = 2.5 Hz), 164.1 (d, *J* = 255.7 Hz), 164.0, 140.2, 131.5 (d, *J* = 8.3 Hz), 128.7 (d, *J* = 7.4 Hz), 124.3 (d, *J* = 22.3 Hz), 120.2 (d, *J* = 24.8 Hz), 118.7, 108.5 (d, *J* = 2.5 Hz), 98.2, 56.0, 55.2.

### 3.3. General Procedure for the Synthesis of Isocoumarin ***3***

To a screw-capped reaction tube were added the β-ketoester (0.10 mmol), diethyl ether (1.0 mL, 0.1 M), and cesium carbonate (0.20 mmol). The resulting mixture was stirred at room temperature for 10 min, after which the diaryliodonium salt (0.20 mmol) was added. The reaction mixture was then stirred at room temperature for 20 h. Upon completion, the crude mixture was purified by column chromatography on silica gel, eluting with 10–20% ethyl acetate in *n*-hexane, to afford the corresponding isocoumarin product.

Methyl 3-methyl-1-oxo-1H-isochromene-4-carboxylate (**3aa**). White amorphous solid (16.9 mg, 77%). M.p. 96–97 °C. ^1^H NMR (400 MHz, CDCl_3_): *δ* 8.28 (d, *J* = 8.3 Hz, 1H, Ar–H), 7.76–7.71 (m, 2H, Ar–H), 7.52–7.48 (m, 1H, Ar–H), 3.97 (s, 3H, CO_2_Me), 2.45 (s, 3H, Me). ^13^C{^1^H} NMR (101 MHz, CDCl_3_): *δ* 166.4, 161.3, 158.2, 135.3, 134.7, 1298, 128.3, 124.3 119.6, 110.1, 52.6, 19.5. These data are consistent with the previously reported results [75].

Methyl 3-methyl-1-oxo-6-(trifluoromethyl)-1H-isochromene-4-carboxylate (**3ba**). White amorphous solid (22.0 mg, 74%). M.p. 102–103 °C. ^1^H NMR (400 MHz, CDCl_3_): *δ* 8.40 (d, *J* = 8.3 Hz, 1H, Ar–H), 8.13 (s, 1H. Ar–H), 7.73 (d, *J* = 8.3 Hz, 1H, Ar–H), 4.00 (s, 3H, CO_2_Me), 2.51 (s, 3H, Me). ^13^C{^1^H} NMR (101 MHz, CDCl_3_): *δ* 165.7, 160.5, 160.0, 136.6 (q, *J* = 33.1 Hz), 135.2, 130.7, 124.6 (q, *J* = 3.6 Hz), 123.4 (q, *J* = 273.4 Hz), 121.9 (q, *J* = 4.1 Hz), 109.4, 52.7, 19.9. ^19^F NMR (376 MHz, CDCl_3_): *δ* −63.0 ppm. IR (KBr): 2962 (C–H), 1768 (C=O), 1718 (C=O), 1181 (C–O) cm^−1^. HRMS-DART (*m*/*z*): [M^+^] Calcd for C_13_H_9_F_3_O_4_^+^ 286.0453; Found 286.0455.

Methyl 3-methyl-6-nitro-1-oxo-1H-isochromene-4-carboxylate (**3ca**). Reddish yellow amorphous solid (19.9 mg, 72%). M.p. 167–168 °C. ^1^H NMR (400 MHz, CDCl_3_): *δ* 8.76 (d, *J* = 2.0 Hz, 1H, Ar–H), 8.45 (d, *J* = 8.8 Hz, 1H, Ar–H), 8.27 (dd, *J* = 8.8, 2.0 Hz, 1H, Ar–H), 4.03 (s, 3H, CO_2_Me), 2.54 (s, 3H, Me). ^13^C{^1^H} NMR (101 MHz, CDCl_3_): *δ* 165.4, 161.7, 159.5, 152.0, 136.1, 131.7, 123.5, 122.3, 120.3, 109.2, 53.0, 20.1. IR (KBr): 2933 (C–H), 1758 (C=O), 1717 (C=O), 1540 (NO_2_) cm^−1^. HRMS-DART (*m*/*z*): [M^+^] Calcd for C_12_H_9_NO_6_ 263.0430; Found 263.0427.

Dimethyl 3-methyl-1-oxo-1H-isochromene-4,6-dicarboxylate (**3da**). White amorphous solid (19.3 mg, 68%). M.p. 155–156 °C. ^1^H NMR (400 MHz, CDCl_3_): *δ* 8.44 (s, 1H, Ar–H), 8.34 (d, *J* = 8.3 Hz, 1H, Ar–H), 8.11 (dd, *J* = 8.3, 1.0 Hz, 1H, Ar–H), 4.00 (s, 3H, CO_2_Me), 3.97 (s, 3H, CO_2_Me), 2.48 (s, 3H, Me). ^13^C{^1^H} NMR (101 MHz, CDCl_3_): *δ* 166.0, 165.9, 160.5, 159.3, 136.0, 134.8, 130.1, 128.6, 126.0, 122.5, 109.9, 52.9, 52.8, 19.7. IR (KBr): 2967 (C–H), 1717 (C=O), 1295 (C-O), 1061 (C–O) cm^−1^. HRMS-DART (*m*/*z*): [M^+^] Calcd for C_14_H_12_O_6_^+^ 276.0634; Found 276.0637.

Dimethyl 3-methyl-1-oxo-1H-isochromene-4,5-dicarboxylate (**3ea**). White amorphous solid (9.2 mg, 31%). M.p. 108–109 °C. ^1^H NMR (400 MHz, CDCl_3_): *δ* 8.43 (dd, *J* = 7.8, 1.5 Hz, 1H, Ar–H), 8.12 (dd, *J* = 7.8, 1.5 Hz, 1H, Ar–H), 7.56 (t, *J* = 7.8 Hz, 1H, Ar–H), 3.89 (s, 3H, CO_2_Me), 3.81 (s, 3H, CO_2_Me), 2.52 (s, 3H, Me). ^13^C{^1^H} NMR (101 MHz, CDCl_3_): *δ* 167.6, 166.2, 160.4, 159.6, 136.4, 133.7, 132.8, 128.7, 127.7, 121.1, 109.2, 52.4, 52.0, 19.1. IR (KBr): 2953 (C–H), 1730 (C=O), 1623 (C=C), 1448 (C=C) cm^−1^. HRMS-DART (*m*/*z*): [M^+^] Calcd for C_14_H_12_O_6_^+^ 276.0634; Found 276.0632.

Methyl 7-fluoro-3-methyl-1-oxo-1H-isochromene-4-carboxylate (**3fa**). White amorphous solid (16.1 mg, 69%). M.p. 112–113 °C. ^1^H NMR (400 MHz, CDCl_3_): *δ* 7.94 (dd, *J* = 8.3, 2.4 Hz, 1H, Ar–H), 7.85 (dd, *J* = 8.3, 4.9 Hz, 1H, Ar–H), 7.49–7.44 (m, 1H, Ar–H), 3.97 (s, 3H, CO_2_Me), 2.47 (s, 3H, Me). ^13^C{^1^H} NMR (101 MHz, CDCl_3_): *δ* 166.0, 161.6 (d, *J* = 250.8 Hz), 160.2 (d, *J* = 3.3 Hz), 158.0 (d, *J* = 2.5 Hz), 131.1 (d, *J* = 3.3 Hz), 127.1 (d, *J* = 7.4 Hz), 123.3 (d, *J* = 22.3 Hz), 121.2 (d, *J* = 8.3 Hz), 115.0 (d, *J* = 23.2 Hz), 109.3, 52.6, 19.4. ^19^F NMR (376 MHz, CDCl_3_): *δ* −107.5 ppm. IR (KBr): 3082 (C–H), 2926 (C–H), 1727 (C=O), 1498 (C=C), 1096 (C–F) cm^−1^. HRMS-DART (*m*/*z*): [M^+^] Calcd for C_12_H_9_FO_4_^+^ 236.0485; Found 236.0483.

Methyl 7-bromo-3-methyl-1-oxo-1H-isochromene-4-carboxylate (**3ga**). White amorphous solid (17.5 mg, 61%). M.p. 111–112 °C. ^1^H NMR (400 MHz, CDCl_3_): *δ* 8.39 (d, *J* = 2.2 Hz, 1H, Ar–H), 7.81 (dd, *J* = 8.8, 2.2 Hz, 1H, Ar–H), 7.69 (d, *J* = 8.8 Hz, 1H, Ar–H), 3.96 (s, 3H, CO_2_Me), 2.45 (s, 3H, Me). ^13^C{^1^H} NMR (101 MHz, CDCl_3_): *δ* 166.0, 159.9, 159.2, 138.3, 133.5, 132.2, 126.3, 122.0, 121.1, 109.5, 52.7, 19.8. IR (KBr): 3077 (C–H), 2953 (C–H), 1747 (C=O), 1618 (C=C), 1243 (C–O) cm^−1^. HRMS-DART (*m*/*z*): [M^+^] Calcd for C_12_H_9_BrO_4_^+^ 295.9684; Found 295.9685.

Methyl 6-chloro-3-methyl-1-oxo-1H-isochromene-4-carboxylate (**3ha**). White amorphous solid (18.2 mg, 67%). M.p. 145–146 °C. ^1^H NMR (400 MHz, CDCl_3_): *δ* 8.18 (d, *J* = 8.6 Hz, 1H, Ar–H), 7.80 (d, *J* = 1.7 Hz, 1H, Ar–H), 7.45 (dd, *J* = 8.6, 1.7 Hz, 1H, Ar–H), 3.97 (s, 3H, CO_2_Me), 2.46 (s, 3H, Me). ^13^C{^1^H} NMR (101 MHz, CDCl_3_): *δ* 165.79, 160.35, 160.19, 142.17, 135.99, 131.31, 128.80, 124.29, 117.80, 109.08, 52.67, 19.84. IR (KBr): 2959 (C–H), 1772 (C=O), 1724 (C=O), 1059 (C–O) cm^−1^. HRMS-DART (*m*/*z*): [M^+^] Calcd for C_12_H_9_ClO_4_^+^ 252.0189; Found 252.0188.

Methyl 3,7-dimethyl-1-oxo-1H-isochromene-4-carboxylate (**3ia**). White amorphous solid (6.3 mg, 29%). M.p. 119–120 °C. ^1^H NMR (400 MHz, CDCl_3_): *δ* 8.09 (d, *J* = 1.5 Hz, 1H, Ar–H), 7.67 (d, *J* = 8.3 Hz, 1H, Ar–H), 7.55 (dd, *J* = 8.3, 1.5 Hz, 1H, Ar–H), 3.96 (s, 3H, CO_2_Me), 2.46 (s, 3H, Me), 2.44 (s, 3H, Me). ^13^C{^1^H} NMR (101 MHz, CDCl_3_): *δ* 166.6, 161.5, 157.5, 138.7, 136.5, 132.2, 129.5, 124.3, 119.5, 110.0, 52.5, 21.3, 19.5. These data are consistent with the previously reported results [75].

Methyl 3-methyl-1-oxo-1H-benzo[g]isochromene-4-carboxylate (**3ja**). Pale yellow amorphous solid (6.1 mg, 21%). M.p. 138–139 °C. ^1^H NMR (400 MHz, CDCl_3_): *δ* 8.93 (s, 1H, Ar–H), 8.18 (s, 1H, Ar–H), 8.02 (d, *J* = 8.3 Hz, 1H, Ar–H), 7.94 (d, *J* = 8.3 Hz, 1H, Ar–H), 7.65 (t, *J* = 7.3 Hz, 1H, Ar–H), 7.57 (t, *J* = 7.3 Hz, 1H, Ar–H), 4.04 (s, 3H, CO_2_Me), 2.47 (s, 3H, Me). ^13^C{^1^H} NMR (101 MHz, CDCl_3_): *δ* 166.8, 161.6, 156.5, 132.3, 129.8, 129.6, 129.1, 128.5, 127.3, 123.2, 117.8, 110.1, 52.7, 19.6. IR (KBr): 2992 (C–H), 1718 (C=O), 1660 (C=O), 1440 (C=C), 1260 (C–O) cm^−1^. HRMS-DART (*m*/*z*): [M^+^] Calcd for C_16_H_12_O_4_^+^ 268.0736; Found 268.0734.

Methyl 5-methyl-7-oxo-7H-thieno[2,3-c]pyran-4-carboxylate (**3ka**). Yellow amorphous solid (14.1 mg, 62%). M.p. 129–130 °C. ^1^H NMR (400 MHz, CDCl_3_): *δ* 7.85 (d, *J* = 4.9 Hz, 1H, Ar–H), 7.70 (d, *J* = 5.4 Hz, 1H, Ar–H), 3.95 (s, 3H, CO_2_Me), 2.65 (s, 3H, Me). ^13^C{^1^H} NMR (101 MHz, CDCl_3_): *δ* 165.5, 164.8, 157.1, 145.2, 136.8, 126.4, 122.3, 108.0, 52.4, 20.2. IR (KBr): 3108 (C–H), 2961 (C–H), 1746 (C=O), 1715 (C=O), 1423 (C=C) cm^−1^. HRMS-DART (*m*/*z*): [M^+^] Calcd for C_10_H_8_O_4_S^+^ 224.0143; Found 224.0140.

Methyl 1-imino-3-methyl-1H-isochromene-4-carboxylate (**3la**). Pale yellow amorphous solid (5.4 mg, 23%). M.p. 80–81 °C. ^1^H NMR (400 MHz, CDCl_3_): *δ* 13.10 (s, 1H, NH), 7.65 (d, *J* = 7.3 Hz, 1H, Ar–H), 7.54 (t, *J* = 7.3 Hz, 1H, Ar–H), 7.37 (t, *J* = 7.3 Hz, 1H, Ar–H), 7.25 (d, *J* = 7.3 Hz, 1H, Ar–H), 3.67 (s, 3H, CO_2_Me), 1.82 (s, 3H, Me). ^13^C{^1^H} NMR (101 MHz, CDCl_3_): *δ* 175.7, 171.8, 139.2, 132.9, 132.7, 132.6, 128.0, 118.0, 115.3, 101.4, 52.2, 19.9. IR (KBr): 3064 (C–H), 1609 (C=N),1443 (C=C), 1263 (C–O) cm^−1^. HRMS-DART (*m*/*z*): ([M+]) Calcd for C_12_H_11_NO_3_^+^ 217.0739; Found 217.0736.

Ethyl 3-methyl-1-oxo-1H-isochromene-4-carboxylate (**3ab**). Pale yellow oil (15.9 mg, 69%). ^1^H NMR (400 MHz, CDCl_3_): *δ* 8.27 (d, *J* = 7.3 Hz, 1H, Ar–H), 7.77–7.70 (m, 2H, Ar–H), 7.52–7.48 (m, 1H, Ar–H), 4.44 (q, *J* = 7.1 Hz, 2H, CH_2_), 2.45 (s, 3H, Me), 1.42 (t, *J* = 7.1 Hz, 3H, Me). ^13^C{^1^H} NMR (101 MHz, CDCl_3_): *δ* 165.9, 161.3, 157.8, 135.2, 134.7, 129.8, 128.3, 124.2, 119.6, 110.3, 61.8, 19.4, 14.3. These data are consistent with the previously reported results [75].

*tert*-Butyl 3-methyl-1-oxo-1H-isochromene-4-carboxylate (**3ac**). Pale yellow oil (10.6 mg, 40%). ^1^H NMR (400 MHz, CDCl_3_): *δ* 8.28 (d, *J* = 8.3 Hz, 1H, Ar–H), 7.73–7.71 (m, 2H, Ar–H), 7.50–7.49 (m, 1H, Ar–H), 2.43 (s, 3H, Me), 1.63 (s, 9H, 3 x Me). ^13^C{^1^H} NMR (101 MHz, CDCl_3_): *δ* 165.2, 161.6, 156.4, 135.2, 135.0, 129.8, 128.2, 124.0, 119.7, 111.8, 83.2, 28.4, 19.1. These data are consistent with the previously reported results [76].

Allyl 3-methyl-1-oxo-1H-isochromene-4-carboxylate (**3ad**). Pale yellow oil (7.8 mg, 32%). ^1^H NMR (400 MHz, CDCl_3_): *δ* 8.29 (d, *J* = 7.3 Hz, 1H, Ar–H), 7.76–7.74 (m, 2H, Ar–H), 7.54–7.50 (m, 1H. Ar–H), 6.10–6.00 (m, 1H, =CH), 5.45 (dd, *J* = 17.1, 1.3 Hz, 1H, =CH), 5.36 (dd, *J* = 10.5, 1.3 Hz, 1H, =CH), 4.88 (dt, *J* = 3.7, 2.1 Hz, 2H, CH_2_), 2.47 (s, 3H, Me). ^13^C{^1^H} NMR (101 MHz, CDCl_3_): *δ* 165.7, 161.3, 158.2, 135.3, 134.7, 131.4, 129.9, 128.4, 124.3, 120.0, 119.6, 110.2, 66.5, 19.6. These data are consistent with the previously reported results [76].

Methyl 3-ethyl-1-oxo-1H-isochromene-4-carboxylate (**3ae**). Pale yellow oil (8.1 mg, 36%).^1^H NMR (400 MHz, CDCl_3_): *δ* 8.30 (d, *J* = 8.3 Hz, 1H, Ar–H), 7.76–7.72 (m, 1H, Ar–H), 7.69 (d, *J* = 7.7 Hz, 1H, Ar–H), 7.52 (t, *J* = 7.7 Hz, 1H, Ar–H), 3.98 (s, 3H, CO_2_Me), 2.71 (q, *J* = 7.5 Hz, 2H, CH_2_), 1.33 (t, *J* = 7.3 Hz, 3H, Me). ^13^C{^1^H} NMR (101 MHz, CDCl_3_): *δ* 166.4, 161.9, 161.5, 135.2, 134.6, 129.8, 128.3, 124.2, 119.6, 109.6, 52.6, 26.5, 12.3. IR (KBr): 2940 (C–H), 1750 (C=O), 1626 (C=C), 1488 (C=C) cm^−1^. HRMS-DART (*m*/*z*): [M^+^] Calcd for C_13_H_12_O_4_^+^ 232.0736; Found 232.0740.

### 3.4. Derivatization of Isocoumarin ***3aa***

3-Methyl-1-oxo-1H-isochromene-4-carbonitrile (**4**). To a screw-capped reaction tube were added isocoumarin **3aa** (21.2 mg, 0.10 mmol), iodine (11.0 mg, 0.09 mmol), phosphorus trichloride (7 μL, 0.08 mmol), and MeCN (2.0 mL, 0.05 M). The reaction mixture was stirred at 160 °C for 36 h, after which the solvent was removed under reduced pressure. The resulting crude residue was quenched with an aqueous solution of Na_2_S_2_O_3_, and the aqueous phase was extracted with EtOAc (3 ×). The combined organic extracts were washed with brine, dried over Na_2_SO_4_, filtered, and concentrated under reduced pressure. The crude product was purified by column chromatography on silica gel, eluting with 15% EtOAc in *n*-hexane, to afford the desired product as a white amorphous solid (14.5 mg, 81%). M.p. 124–125 °C. ^1^H NMR (400 MHz, CDCl_3_): *δ* 8.29 (d, *J* = 8.5 Hz, 1H, Ar–H), 7.86 (t, *J* = 7.6 Hz, 1H, Ar–H), 7.71 (d, *J* = 8.5 Hz, 1H, Ar–H), 7.61 (t, *J* = 7.6 Hz, 1H, Ar–H), 2.61 (s, 3H, Me). ^13^C{^1^H} NMR (101 MHz, CDCl_3_): *δ* 165.5, 159.7, 136.2, 133.4, 130.2, 129.7, 123.8, 118.7, 114.3, 92.9, 20.0. These data are consistent with the previously reported results [77].

Methyl 3-methyl-1-oxo-1,2-dihydroisoquinoline-4-carboxylate (**5**). To a screw-capped reaction tube were added isocoumarin **3aa** (23.9 mg, 0.11 mmol), NH_4_OAc (23.1 mg, 0.30 mmol), and DMSO (2.0 mL, 0.055 M). The reaction mixture was stirred at 100 °C for 3 h, after which it was diluted with water and extracted with EtOAc (3 ×). The combined organic extracts were washed with brine, dried over Na_2_SO_4_, and concentrated under reduced pressure. The crude residue was purified by column chromatography on silica gel, eluting with 15% EtOAc in *n*-hexane, to afford the desired product as a white amorphous solid (11.5 mg, 48%). M.p. 233–234 °C. ^1^H NMR (400 MHz, DMSO-d_6_): *δ* 11.68 (s, 1H, NH), 8.19 (dd, *J* = 7.8, 1.0 Hz, 1H, Ar–H), 7.79 (d, *J* = 8.3 Hz, 1H, Ar–H), 7.72 (td, *J* = 7.6, 1.5 Hz, 1H, Ar–H), 7.49 (t, *J* = 7.6 Hz, 1H, Ar–H), 3.87 (s, 3H, CO_2_Me), 2.34 (s, 3H, Me). ^13^C{^1^H} NMR (101 MHz, DMSO-d_6_): *δ* 167.0, 161.7, 142.2, 135.0, 133, 126.8, 126.2, 124.1, 123.9, 107.1, 52.1, 18.1. These data are consistent with the previously reported results [78].

Methyl 3-(bromomethyl)-1-oxo-1H-isochromene-4-carboxylate (**6**). To a screw-capped reaction tube were added isocoumarin **3aa** (663.6 mg, 3.0 mmol), *N*-bromosuccinimide (635.6 mg, 3.6 mmol), benzoyl peroxide (74.7 mg, 0.30 mmol), and dimethyl carbonate (2.0 mL, 1.5 M). The reaction mixture was stirred at 80 °C for 4 h, after which the solvent was removed under reduced pressure. The crude residue was purified by column chromatography on silica gel, eluting with 15% ethyl acetate in *n*-hexane, to afford the desired product as a white amorphous solid (618.4 mg, 68%). M.p. 108–109 °C. ^1^H NMR (400 MHz, CDCl_3_): *δ* 7.73 (d, *J* = 7.8 Hz, 1H, Ar–H), 7.27–7.23 (m, 2H, Ar–H), 7.04 (t, *J* = 7.3 Hz, 1H, Ar–H), 3.94 (s, 2H, CH_2_), 3.51 (s, 3H, CO_2_Me). ^13^C{^1^H} NMR (101 MHz, CDCl_3_): *δ* 165.2, 160.3, 154.8, 135.5, 133.7, 130.1, 129.7, 125.4, 120.5, 111.8, 53.2, 26.0. IR (KBr): 3078 (C-H), 2957 (C–H), 1742 (C=O), 1714 (C=O), 1613 (C=C), 1086 (C–O) cm^−1^ HRMS-DART (*m*/*z*): ([M+]) Calcd for C_12_H_9_BrO_4_^+^ 295.9684; Found 295.9680 (^79^Br), 297.9663 (^81^Br).

Methyl 1-oxo-3-(phenoxymethyl)-1H-isochromene-4-carboxylate (**7**). To a screw-capped reaction tube were added isocoumarin **6** (57.4 mg, 0.19 mmol), K_2_CO_3_ (57.7 mg, 0.42 mmol), phenol (41.4 mg, 0.44 mmol), and acetone (2.0 mL, 0.095 M). The reaction mixture was stirred at 80 °C for 6 h, then filtered. The filtrate was concentrated under reduced pressure, and the resulting crude residue was purified by column chromatography on silica gel, eluting with 15% EtOAc in *n*-hexane, to afford the desired product as a pale yellow oil (29.7 mg, 50%). M.p. 70–71 °C. ^1^H NMR (400 MHz, CDCl_3_): *δ* 8.25 (d, *J* = 7.8 Hz, 1H, Ar–H), 7.70–7.68 (m, 2H, Ar–H), 7.51 (t, *J* = 6.6 Hz, 1H, Ar–H), 7.22 (t, *J* = 7.8 Hz, 2H, Ar–H), 6.94–6.86 (m, 3H, Ar–H), 4.97 (s, 2H, CH_2_), 3.83 (s, 3H, Me). ^13^C{^1^H} NMR (101 MHz, CDCl_3_): *δ* 165.4, 160.6, 157.9, 154.2, 135.4, 133.8, 130.0, 129.8, 129.4, 124.7, 122.0, 120.3, 114.8, 112.3, 65.8, 53.0. IR (KBr): 3067 (C–H), 2954 (C–H), 1733 (C=O), 1600 (C=C), 1492 (C=C), 1219 (C–O) cm^−1^. HRMS-DART (*m*/*z*): ([M+]) Calcd for C_18_H_14_O_5_^+^ 310.0841; Found 310.0839.

Methyl 3-(azidomethyl)-1-oxo-1H-isochromene-4-carboxylate (**8**). To a screw-capped reaction tube were added isocoumarin **6** (27.5 mg, 0.09 mmol), NaN_3_ (12.0 mg, 0.18 mmol), and dry EtOH (2.0 mL, 0.045 M). The reaction mixture was stirred at room temperature for 48 h, after which EtOAc was added. The organic phase was washed with water and brine, dried over Na_2_SO_4_, filtered, and concentrated under reduced pressure. The crude residue was purified by column chromatography on silica gel, eluting with 15% ethyl acetate in *n*-hexane, to afford the desired product as a white solid (15.5 mg, 65%). M.p. 75–76 °C. ^1^H NMR (400 MHz, CDCl_3_): *δ* 8.32 (d, *J* = 8.3 Hz, 1H, Ar–H), 7.82–7.78 (m, 2H, Ar–H), 7.62–7.58 (m, 1H, Ar–H), 4.35 (s, 2H, CH_2_), 4.01 (s, 3H, Me). ^13^C{^1^H} NMR (101 MHz, CDCl_3_): *δ* 165.0, 160.0, 153.9, 135.3, 133.3, 130.0, 129.5, 125.1, 120.2, 112.0, 52.9, 50.4. IR (KBr): 3079 (C–H), 2108 (N_3_), 1726 (C=C), 1435 (C=C), 1055 (C–O) cm^−1^. HRMS-DART (m/z): [M^+^] Calcd for C_12_H_9_N_3_O_4_^+^ 259.0593; Found 259.0595.

Methyl 3-((diethoxyphosphoryl)methyl)-1-oxo-1H-isochromene-4-carboxylate (**9**). To a screw-capped reaction tube were added isocoumarin **6** (30.6 mg, 0.10 mmol) and P(OEt)_3_ (52 μL, 0.30 mmol). The reaction mixture was stirred at 150 °C for 12 h, after which EtOAc was added. The organic phase was washed with water and brine, dried over Na_2_SO_4_, filtered, and concentrated under reduced pressure. The crude residue was purified by column chromatography on silica gel, eluting with 15% EtOAc in *n*-hexane, to afford the desired product as a pale yellow oil (30.1 mg, 63%). M.p. 74–75 °C. ^1^H NMR (400 MHz, CDCl_3_): *δ* 8.26 (d, *J* = 7.8 Hz, 1H, Ar–H), 7.81 (d, *J* = 8.3 Hz, 1H, Ar–H), 7.73 (dt, *J* = 8.3, 1.3 Hz, 1H, Ar–H), 7.52 (t, *J* = 7.6 Hz, 1H, Ar–H), 4.18–4.07 (m, 4H, 2 × CH_2_), 3.95 (s, 3H, CO_2_Me), 3.52 (d, *J* = 22.4 Hz, 2H, CH_2_), 1.29 (t, *J* = 7.1 Hz, 6H, 2 × Me). ^13^C{^1^H} NMR (101 MHz, CDCl_3_): *δ* 165.5 (d, *J* = 3.3 Hz), 160.5, 152.9 (d, *J* = 13.2 Hz), 135.3, 134.3 (d, *J* = 3.3 Hz), 129.7, 128.8, 124.9, 119.8, 111.6 (d, *J* = 9.9 Hz), 62.8 (d, *J* = 5.8 Hz), 52.7, 31.4 (d, *J* = 135.7 Hz), 16.3 (d, *J* = 6.6 Hz). IR (KBr): 2982 (C–H), 1725 (C=O), 1624 (C=C), 1486 (C=C), 1262 (P=O/C–O) cm^−1^. HRMS-DART (*m*/*z*): [M^+^] Calcd for C_16_H_19_O_7_P^+^ 354.0868; Found 354.0867.

1H-Furo[3,4-c]isochromene-1,5(3H)-dione (**10**). To a screw-capped reaction tube were added isocoumarin **6** (23.5 mg, 0.08 mmol), DMSO (0.17 mL), and water (0.33 mL). The reaction mixture was stirred at 50 °C for 24 h, after which EtOAc was added. The organic phase was washed with water and brine, dried over Na_2_SO_4_, filtered, and concentrated under reduced pressure. The crude residue was purified by column chromatography on silica gel, eluting with 15% EtOAc in *n*-hexane, to afford the desired product as a white solid (11.9 mg, 74%). M.p. 167–168 °C. ^1^H NMR (400 MHz, CDCl_3_): *δ* 8.30 (d, *J* = 7.8 Hz, 1H, Ar–H), 8.25 (d, *J* = 7.8 Hz, 1H, Ar–H), 7.87 (t, *J* = 7.8 Hz, 1H, Ar–H), 7.63 (t, *J* = 7.8 Hz, 1H, Ar–H), 5.08 (s, 2H, CH_2_). ^13^C{^1^H} NMR (101 MHz, CDCl_3_): *δ* 170.4, 167.7, 160.1, 136.4, 131.2, 130.6, 129.9, 123.0, 119.1, 103.1, 66.0. IR (KBr): 3078 (C–H), 2972 (C–H), 1767 (C=O), 1673 (C=O), 1599 (C=C), 1505 (C=C) cm^−1^. HRMS-DART (*m*/*z*): [M^+^] Calcd for C_11_H_6_O_4_^+^ 202.0266; Found 202.0265.

## 4. Conclusions

In summary, a mild, metal-free protocol was developed for the synthesis of diverse isocoumarins via the reaction of *ortho*-carboxylate-ester-substituted diaryliodonium salts with acetoacetates and sequential derivatization. This transformation proceeds via C-arylation of an activated methylene substrate, followed by intramolecular lactonization, culminating in the construction of the isocoumarin framework. A wide range of diaryliodonium salts and acetoacetate esters were effectively employed in this reaction under operationally simple, metal-free conditions. Isocoumarin products featuring synthetically useful functional handles (e.g., halogen, nitro, carboxylate ester, and azide groups) were obtained, facilitating further derivatization and expanding their potential applicability. Moreover, derivatization of the isocoumarin product enabled access to furo[3,4-c]isochromene-1,5-dione, a motif observed in several natural products. Further derivatization and synthesis of key heterocyclic scaffolds are currently underway in our laboratory and will be reported in due course.

## Figures and Tables

**Table 1:** Optimization of the reaction conditions ^a^.

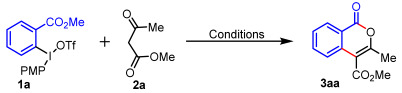					
Entry	Base (Equiv.)	Solvent	Temp.	Time (h)	Yield (%) ^b^
1 ^c^	NaH (1.5)	DMF	rt	72	trace
2 ^c^	NaH (1.5)	DMF	70	6	trace
3 ^d^	Cs_2_CO_3_ (2)	Et_2_O	rt	20	66
4	Cs_2_CO_3_ (2)	Et_2_O	rt	20	64
5 ^e^	Cs_2_CO_3_ (2)	Et_2_O	rt	20	63
6 ^f^	Cs_2_CO_3_ (2)	Et_2_O	rt	20	40
7	Cs_2_CO_3_ (2)	Et_2_O	rt	30	58
8	Cs_2_CO_3_ (2)	Et_2_O	40	20	66
9	K_2_CO_3_ (2)	Et_2_O	rt	20	9
10	*t*-BuOK (2)	Et_2_O	rt	20	9
11	K_3_PO_4_ (2)	Et_2_O	rt	20	9
12	DBU (2)	Et_2_O	rt	20	10
13	Cs_2_CO_3_ (2)	Toluene	rt	20	27
14	Cs_2_CO_3_ (2)	DCM	rt	20	13
15	Cs_2_CO_3_ (2)	THF	rt	20	5
16	Cs_2_CO_3_ (2)	*t*-BuOMe	rt	20	6
17 ^g^	Cs_2_CO_3_ (2)	Et_2_O	rt	20	81 (77) ^h^
18	Cs_2_CO_3_ (2)	Et_2_O/H_2_O	rt	20	ND

^a^ Reaction conditions: **1a** (2.0 equiv), **2a** (0.1 mmol), base (2 equiv), and anhydrous solvent (1.0 mL). ^b 1^H NMR yield using 1,1,2,2-tetrachloroethane as an internal standard. ^c^ **1a** (1.0 equiv) was used. ^d^ 4 Å MS were added. ^e^ Et_2_O (2.0 mL) was used. ^f^ Et_2_O (0.5 mL) was used. ^g^ Et_2_O was used as received without further dehydration. ^h^ Isolated yield.

## Data Availability

The data supporting this work is in the article and Supplementary Materials.

## Supplementary Materials

The following supporting information can be downloaded at https://www.mdpi.com/article/10.3390/molecules31071069/s1, Table S1: optimization of the reaction conditions, effect of dummy ligand and counteranion and ^1^H and ^13^C NMR spectra of the iodonium salts **1e** and **1f**, and the isocoumarin products **3** and their derivatives **4**–**10**.
