# Supplementary material for: Isocoumarin Synthesis via Metal-Free C-Arylation of Acetoacetates with ortho-Ester-Functionalized Diaryliodonium Salts
Source: Molecules. 2026 Mar 24;31(7):1069. doi: 10.3390/molecules31071069 (PMC13074673; doi:10.3390/molecules31071069)

Isocoumarin Synthesis *via* Metal-Free *C*-Arylation of Acetoacetates with *ortho*-Ester-Functionalized Diaryliodonium Salts

Table of contents **Page**

1. Optimization of the reaction conditions 2
2. Effect of dummy ligand and counteranion 4
3. NMR spectra 5

Optimization of the reaction conditions

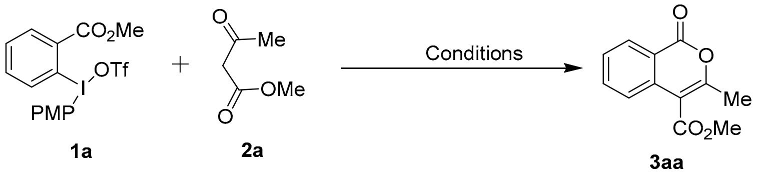

General procedure for the optimization of isocoumarin ***3***

To a screw-capped reaction tube were added the β-ketoester (0.10 mmol), diethyl ether (1.0 mL), and cesium carbonate (0.20 mmol). The resulting mixture was stirred at room temperature for 10 min, after which the diaryliodonium salt (0.20 mmol) was added. The reaction mixture was then stirred at room temperature for 20 h. Upon completion, the mixture was filtered through a cotton plug containing a pad of Na_2SO_4, and the filtrate was concentrated under reduced pressure. The crude reaction mixture was analyzed by ^{1}H NMR to determine the yield using 1,1,2,2-tetrachloroethane as an internal standard.

Table S1: Optimization of the reaction conditions

Entry	Salt (equiv)	Base (equiv)	Solvent	additive	Temp.	Time (h)	Yield (%)[a]
1	1	NaH (1.5)	DMF	---	rt	72	trace
2	1	NaH (1.5)	DMF	---	70	6	trace
3	1	^{t}-BuOK (1.5)	DMF	---	rt	72	trace
4	1	^{t}-BuOK (1.5)	DMF	---	70	6	trace
5	2	Cs_2CO_3 (2)	Et_2O	4 Å MS	rt	20	66
6	1	Cs_2CO_3 (2)	Et_2O	4 Å MS	rt	20	54
7	1	Cs_2CO_3 (1)	Et_2O	4 Å MS	rt	7	35
8	1.5	Cs_2CO_3 (2)	Et_2O	4 Å MS	rt	20	60
9	2	Cs_2CO_3 (3)	Et_2O	4 Å MS	rt	20	62
10	1	Cs_2CO_3 (2)	Et_2O	4 Å MS, 1.5 equiv. of **2**	rt	20	50
11	2	Cs_2CO_3 (2)	Et_2O	---	rt	20	64
12	2	Cs_2CO_3 (2)	Et_2O (2 mL)	---	rt	20	63
13	2	Cs_2CO_3 (2)	Et_2O (0.5 mL)	---	rt	20	40
13	2	Cs_2CO_3 (2)	Et_2O	---	rt	30	58
14	1	Cs_2CO_3 (2)	Et_2O	---	rt	30	50
15	2	Cs_2CO_3 (2)	Et_2O	---	40	9	61
16	1	Cs_2CO_3 (2)	Et_2O	---	40	9	53
17	2	Cs_2CO_3 (2)	Et_2O	---	40	20	66
18	1	Cs_2CO_3 (2)	Et_2O	---	40	20	49

19	2	Na_2CO_3 (2)	Et_2O	---	rt	20	NR
20	2	K_2CO_3 (2)	Et_2O	---	rt	20	9
21	2	Li_2CO_3 (2)	Et_2O	---	rt	20	NR
22	2	NaH (2)	Et_2O	---	rt	20	ND
23	2	t-BuOK (2)	Et_2O	---	rt	20	9
24	2	TEA (2)	Et_2O	---	rt	20	NR
25	2	DBU (2)	Et_2O	---	rt	20	10
26	2	K_3PO_4 (2)	Et_2O	---	rt	20	9
27	2	Cs_2CO_3 (2)	Toluene	---	rt	20	27
28	2	Cs_2CO_3 (2)	DCM	---	rt	20	13
29	2	Cs_2CO_3 (2)	THF	---	rt	20	5
30	2	Cs_2CO_3 (2)	dioxane	---	rt	20	8
31	2	Cs_2CO_3 (2)	MeCN	---	rt	20	Trace
32	2	Cs_2CO_3 (2)	MeOH	---	rt	20	ND
33	2	Cs_2CO_3 (2)	DMF	---	rt	20	10
34	2	Cs_2CO_3 (2)	$^{t-}$BuOMe	---	rt	20	6
35	2	Cs_2CO_3 (2)	AcOEt	---	rt	20	traces
36	2	Cs_2CO_3 (2)	Et_2O	commercial Et_2O	rt	20	81 (77)^b
37	2	Cs_2CO_3 (2)	Et_2O/H_2O (50%)	---	rt	20	ND
38	2	Cs_2CO_3 (2)	Et_2O/H_2O (0.5%)	---	rt	20	30
39	2	Cs_2CO_3 (2)	Et_2O/H_2O (1%)	---	rt	20	30
40	2	Cs_2CO_3 (2)	Et_2O	Add. Na_2SO_4	rt	20	56

(a) ^{1}H-NMR yield with 1,1,2,2-tetrachloroethane as an internal standard. (b) Isolated yield. NR = no rection with recovery of the iodonium salt. ND = not detected with consumption of iodonium salt.

Effect of dummy ligand and counteranion

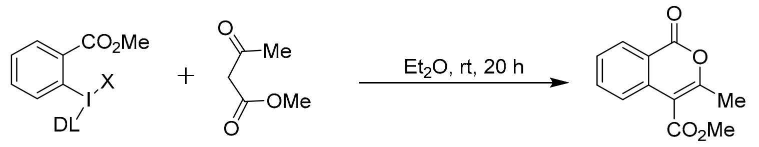

1 2 3

Entry	Dummy Ligand (DL)	Counteranion (X)	Yiled (%)
1	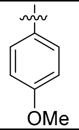	OTf	81
2	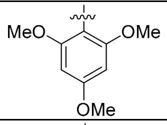	OTs	22
3	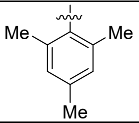	OTf	40
4	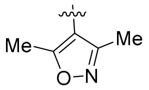	OTf	NR
5		OTs	NR

<u>**Unsuccessful examples of activated methylene nucleophiles**</u>

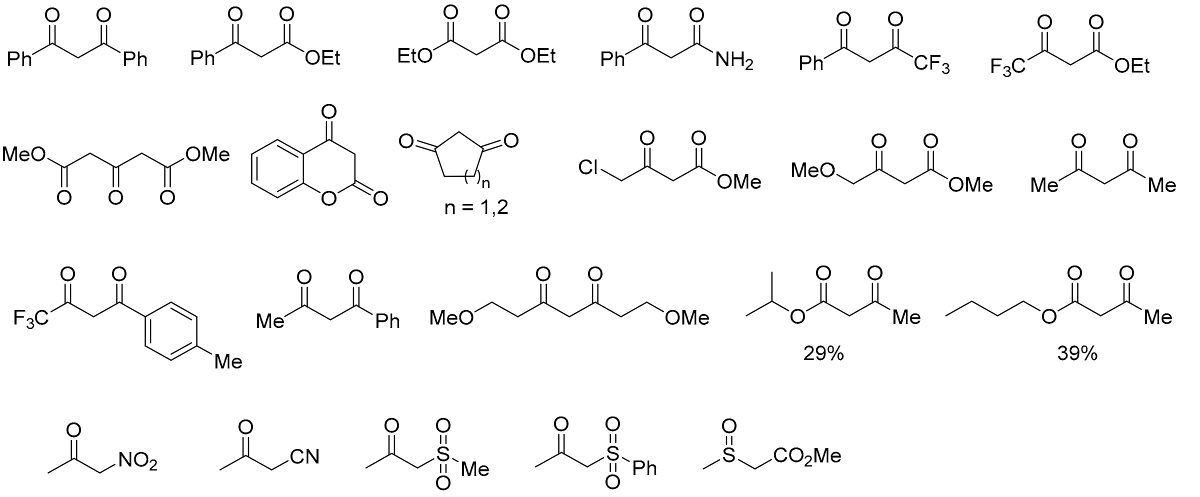

NMR spectra

Dimethyl 2-((4-methoxyphenyl)(((trifluoromethyl)sulfonyl)oxy)-λ³-iodaneyl)isophthalate(1e)

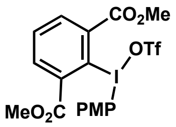

^{1}H NMR (400 MHz, $CDCl_3$ + drop CD_3OD)

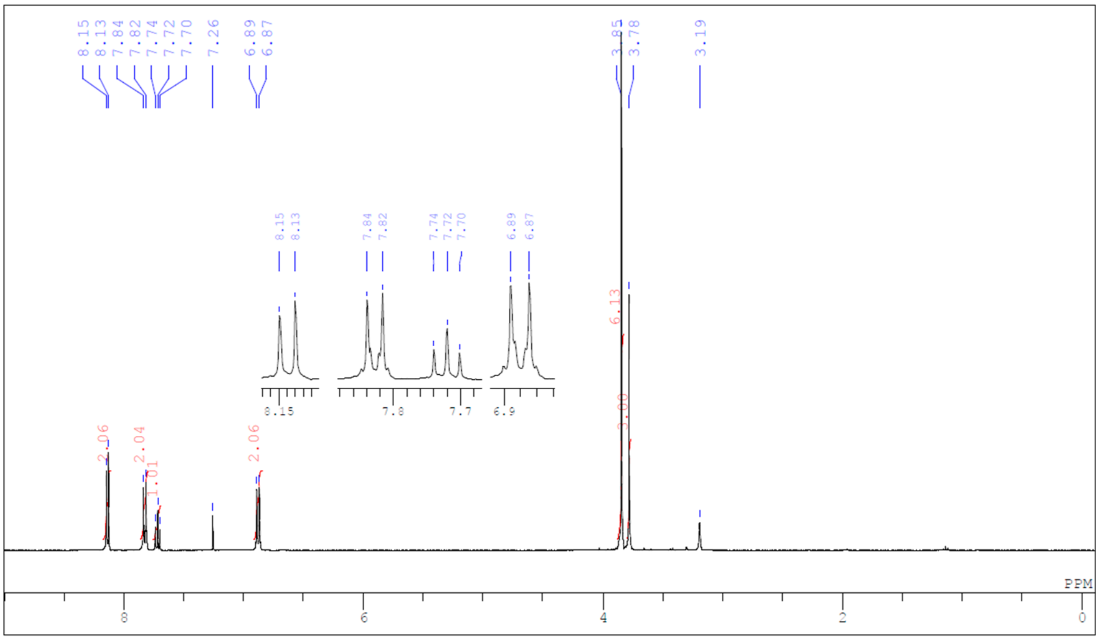

^{13}C{1H} NMR (101 MHz, $CDCl_3$ + drop CD_3OD)

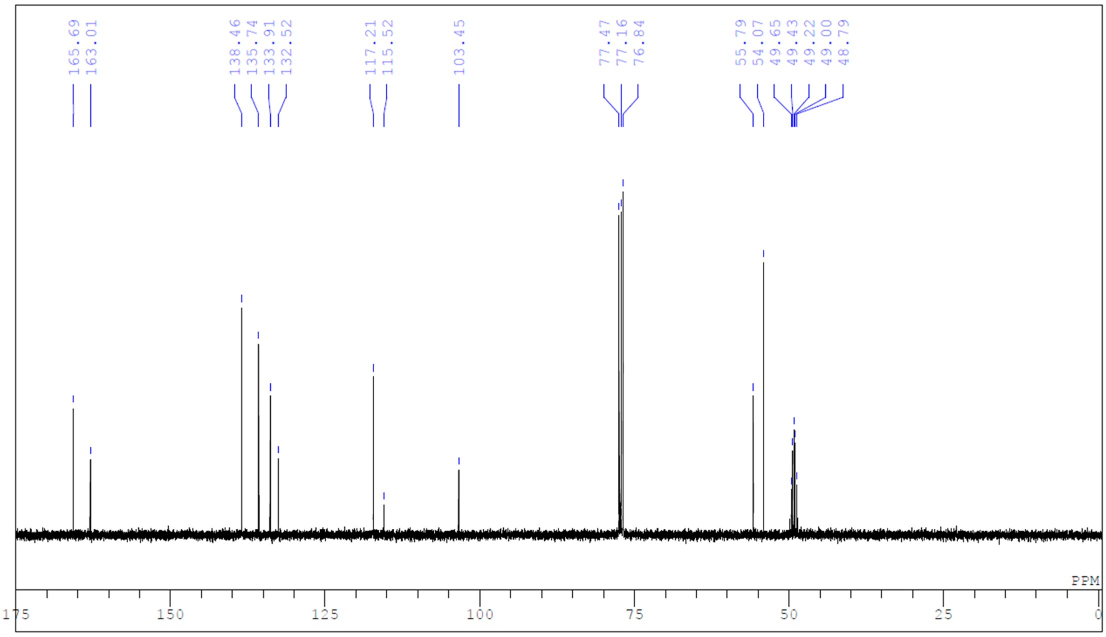

Methyl 5-fluoro-2-((4-methoxyphenyl)(((trifluoromethyl)sulfonyl)oxy)-λ^3-iodaneyl)benzoate (1f)

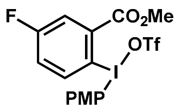

^{1}H NMR (400 MHz, $CDCl_3$)

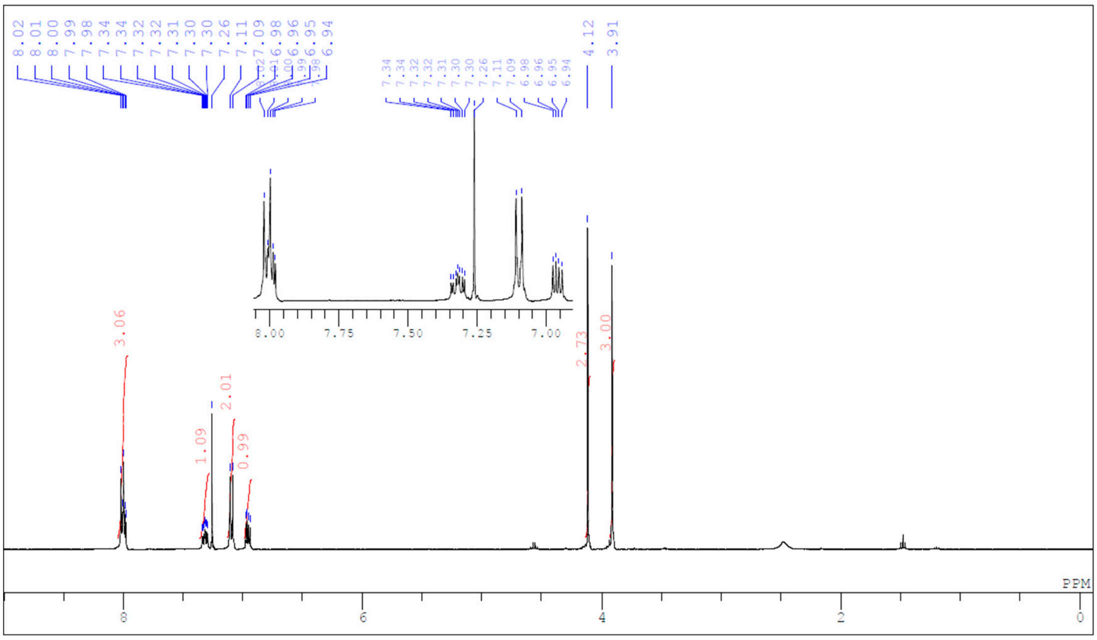

^{13}C{^{1}H} NMR (101 MHz, $CDCl_3$)

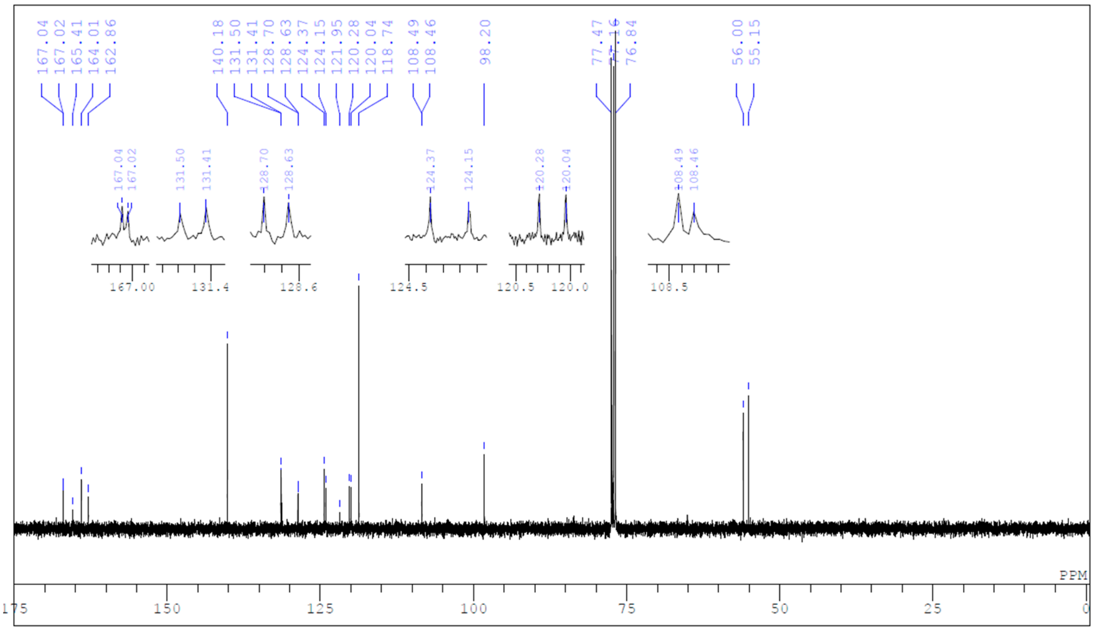

Methyl 3-methyl-1-oxo-1H-isochromene-4-carboxylate (3aa)

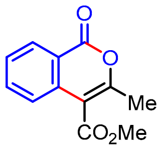

^{1}H NMR (400 MHz, $CDCl_3$)

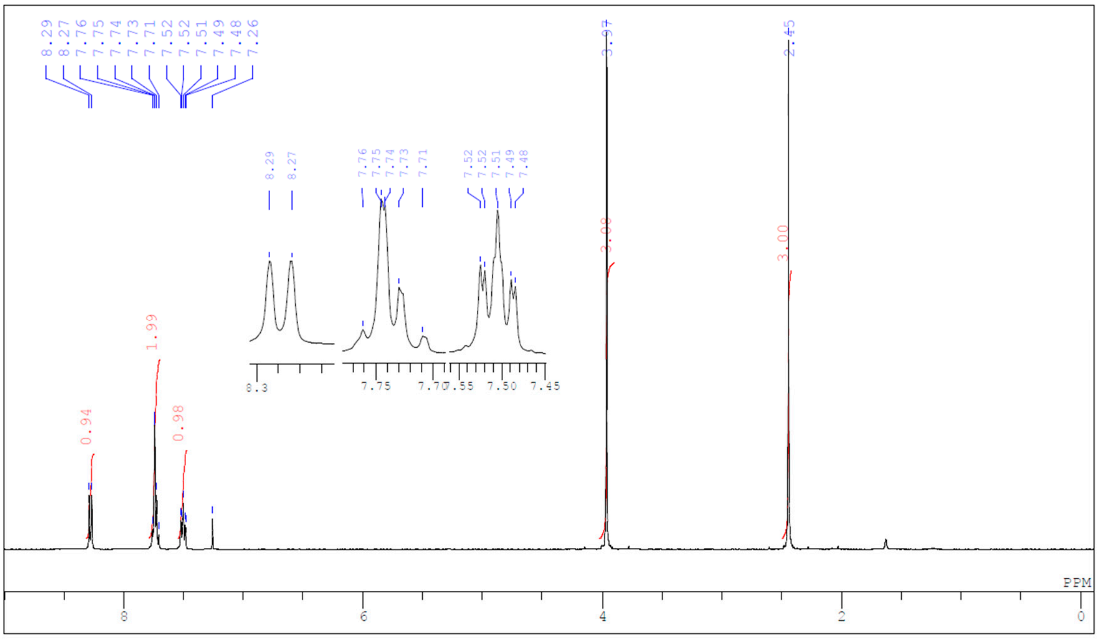

$^{13}C\{1H\}$ NMR ($CDCl_3$)

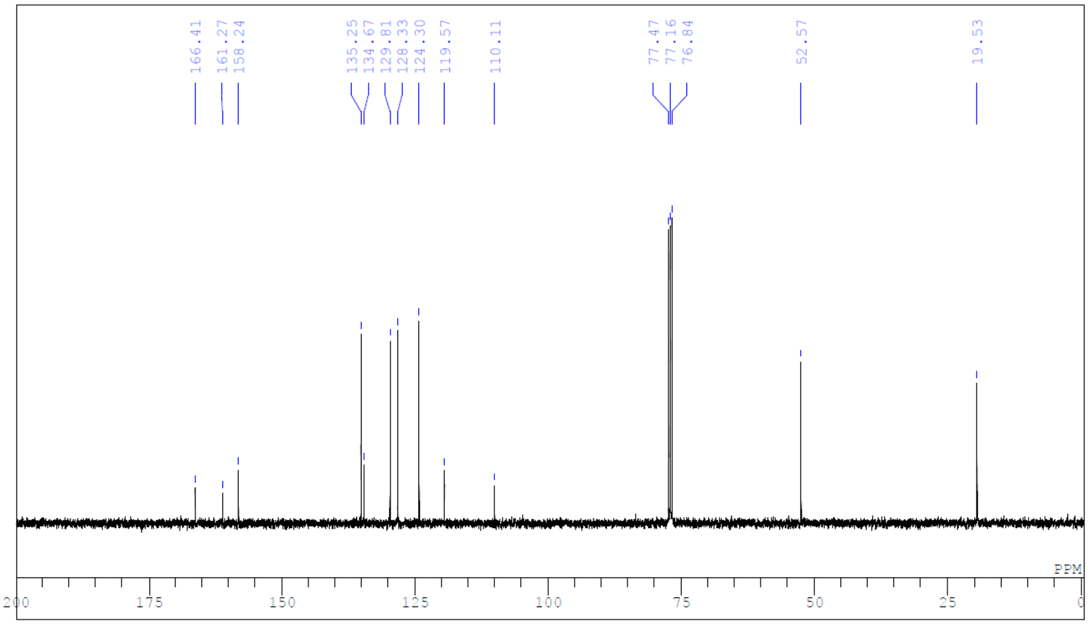

Methyl 3-methyl-1-oxo-6-(trifluoromethyl)-1H-isochromene-4-carboxylate (3ba)

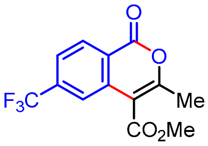

^1H NMR (400 MHz, $CDCl_3$)

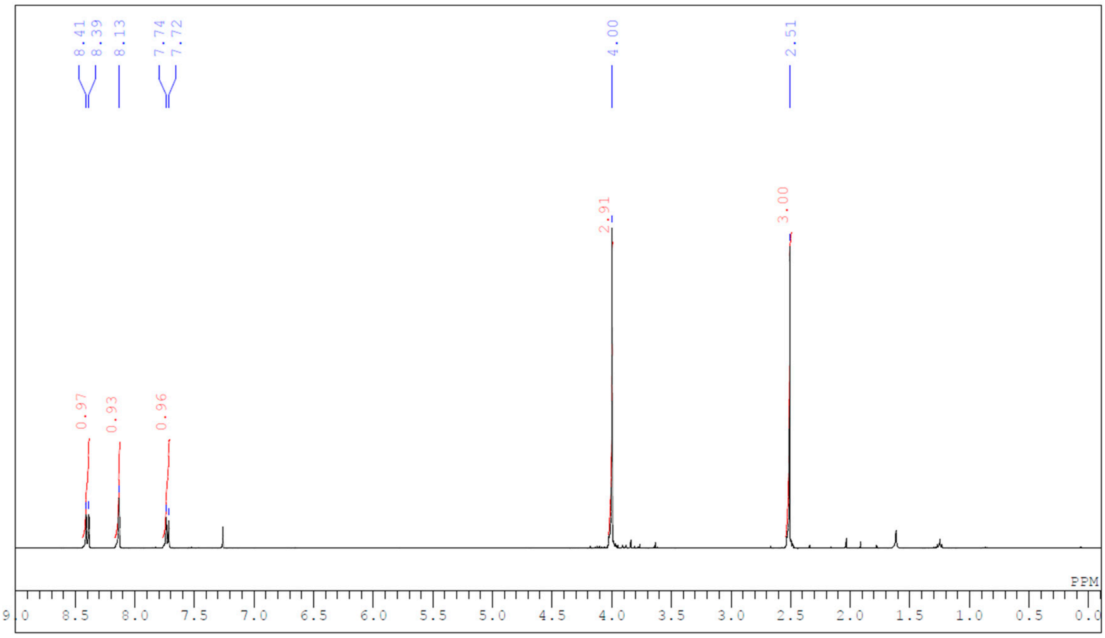

$^{13}C\{^1H\}$ NMR (101 MHz, $CDCl_3$)

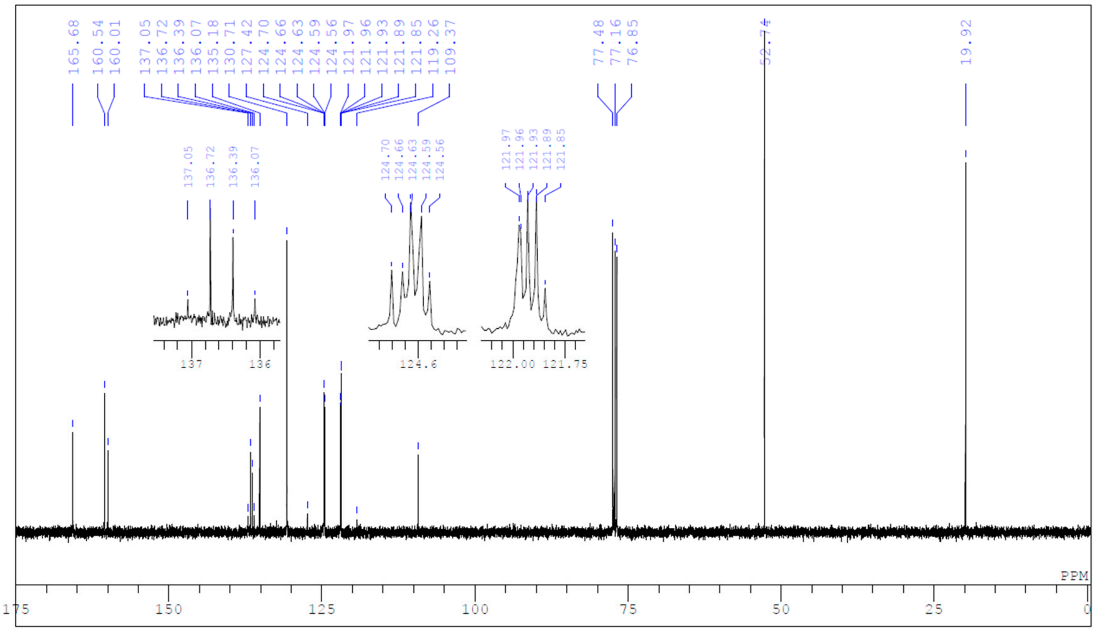

^{19}F NMR (376 MHz, CDCl3) with trifluorotoluene as an internal standard

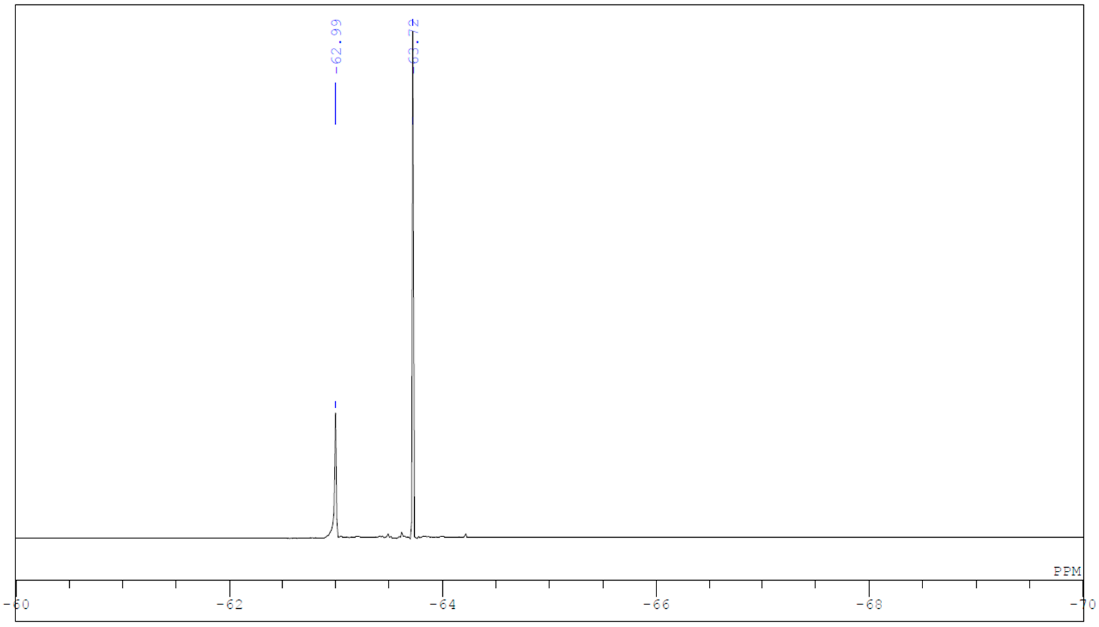

Methyl 3-methyl-6-nitro-1-oxo-1H-isochromene-4-carboxylate (3ca)

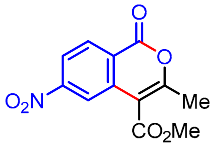

^{1}H NMR (400 MHz, $CDCl_3$)

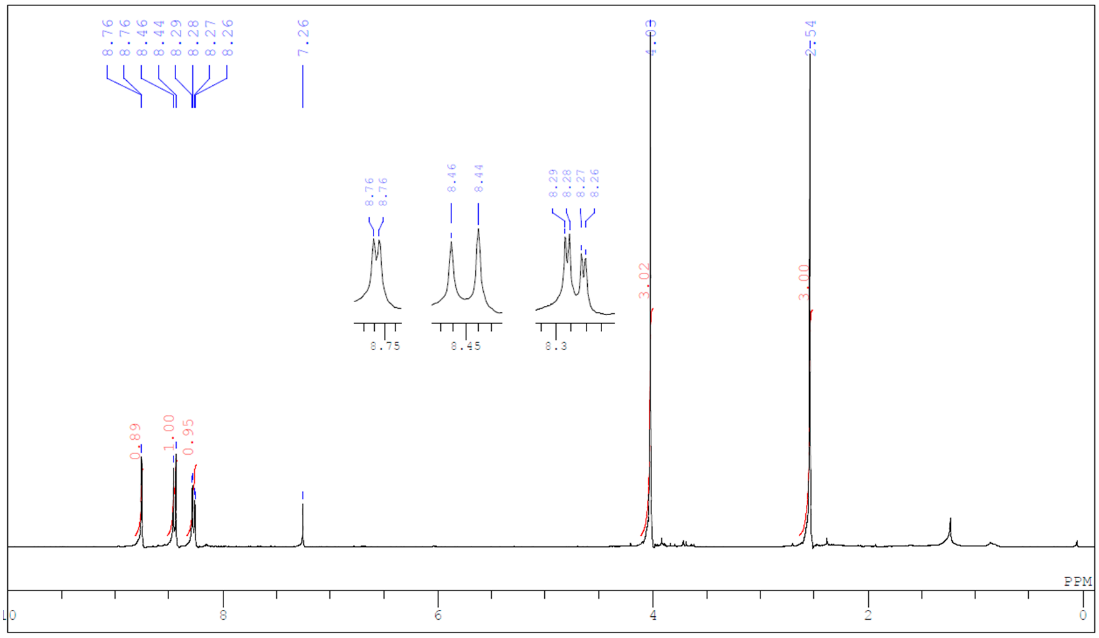

$^{13}C\{^{1}H\}$ NMR (101 MHz, $CDCl_3$)

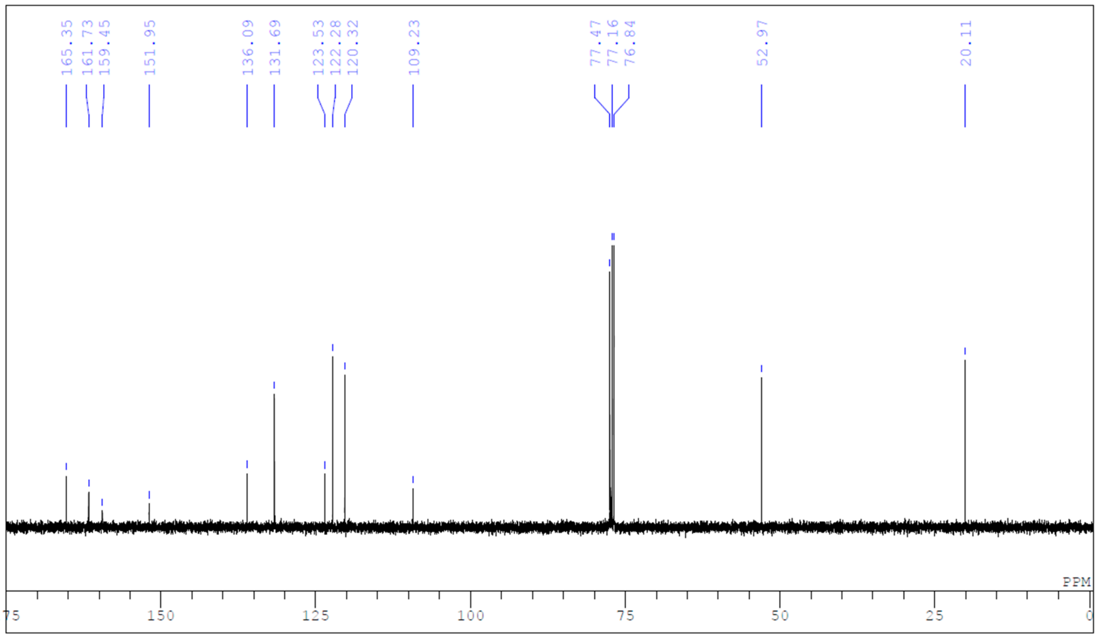

Dimethyl 3-methyl-1-oxo-1H-isochromene-4,6-dicarboxylate (3da)

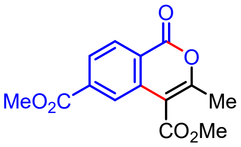

^{1}H NMR (400 MHz, $CDCl_3$)

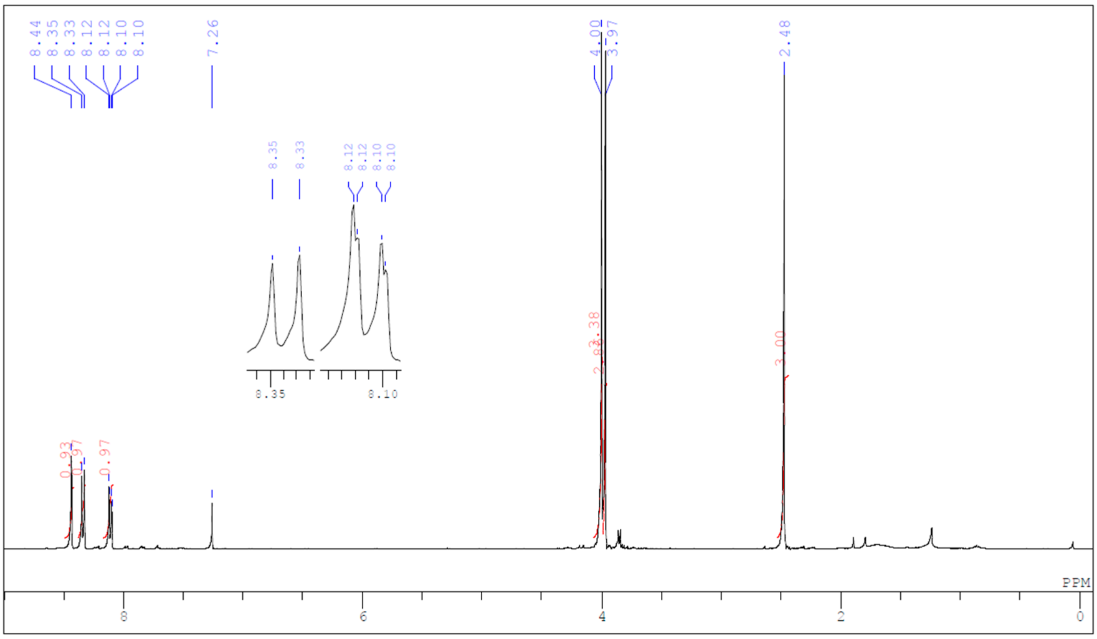

$^{13}C\{^{1}H\}$ NMR (101 MHz, $CDCl_3$)

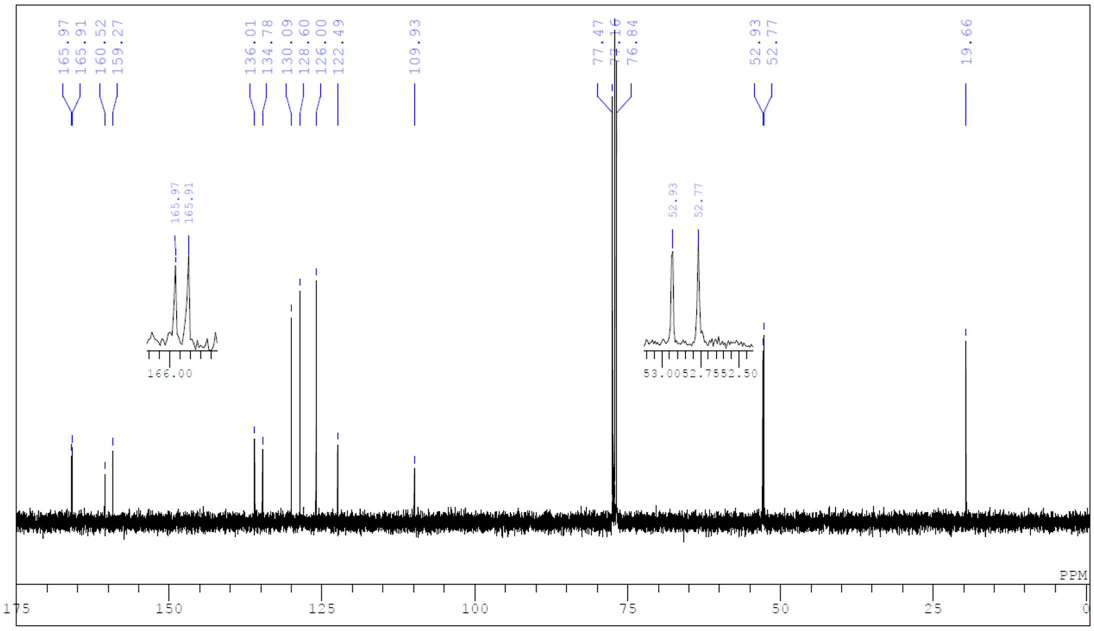

Dimethyl 3-methyl-1-oxo-1H-isochromene-4,5-dicarboxylate (3ea)

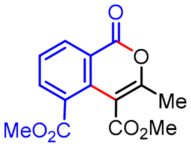

^{1}H NMR (400 MHz, $CDCl_3$)

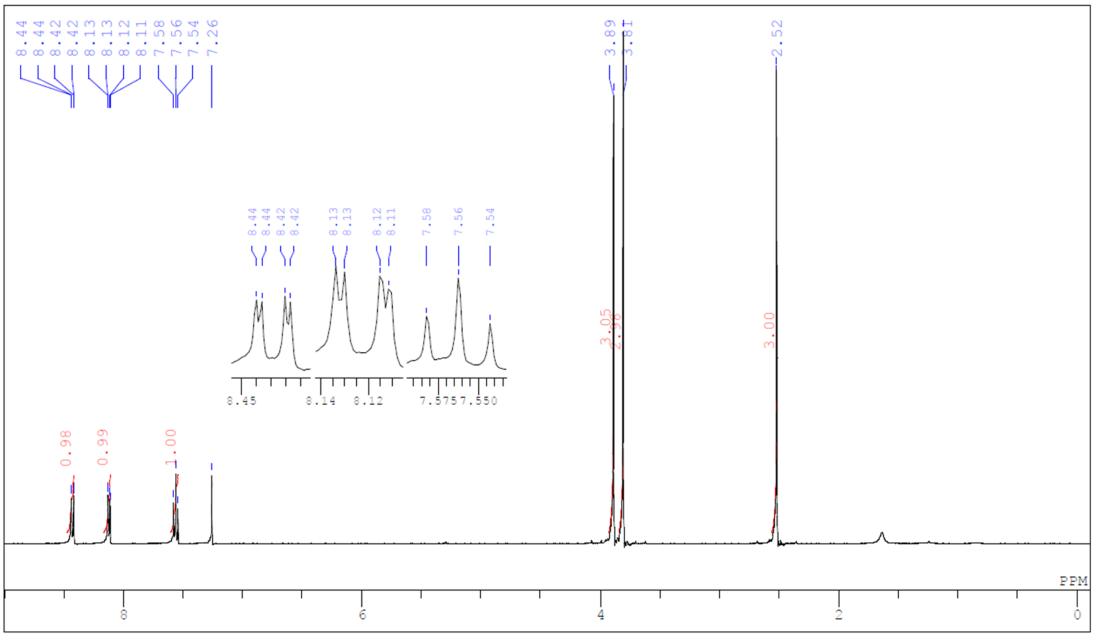

$^{13}C\{^1H\}$ NMR (101 MHz, $CDCl_3$)

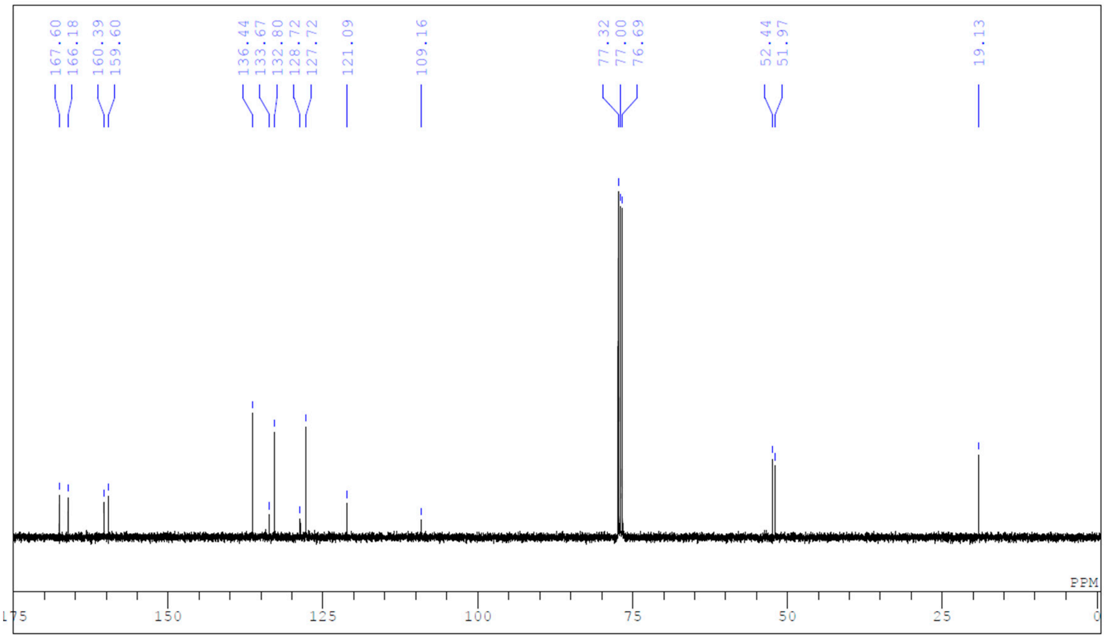

Methyl 7-fluoro-3-methyl-1-oxo-1H-isochromene-4-carboxylate (3fa)

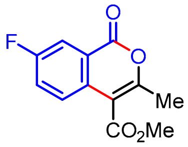

^1H NMR (400 MHz, $CDCl_3$)

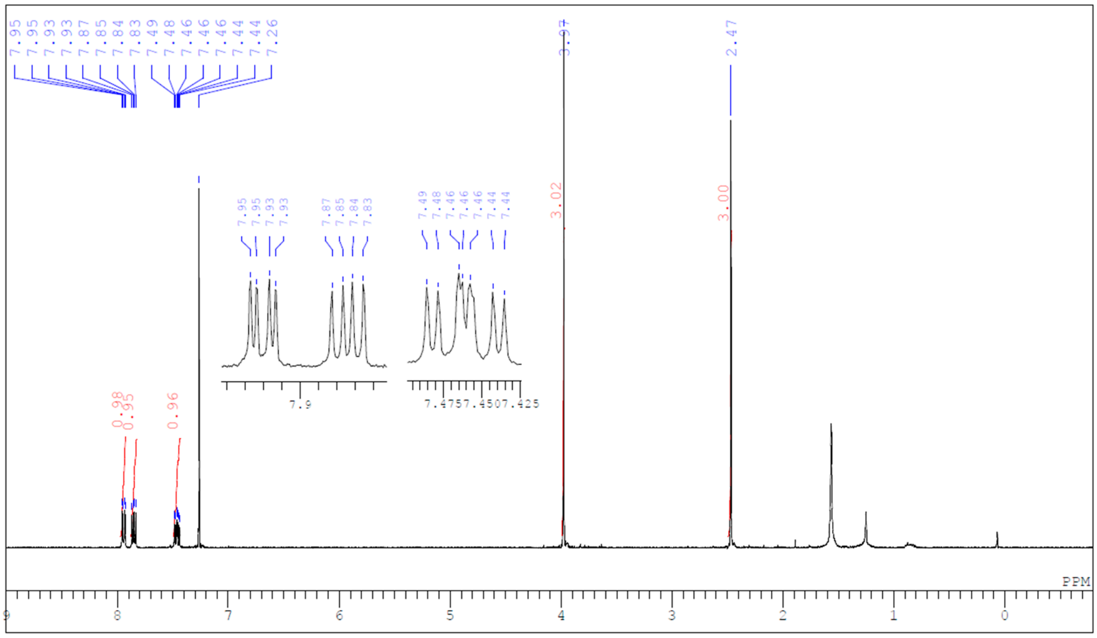

$^{13}C\{1H\}$ NMR ($CDCl_3$)

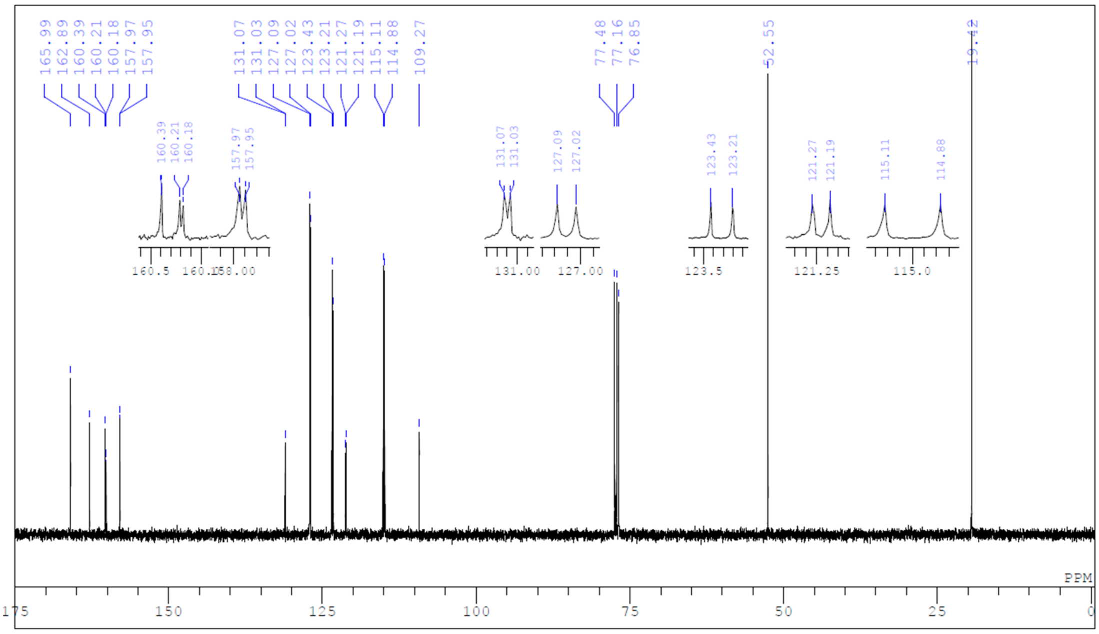

^{19}F NMR (376 MHz, $CDCl_3$) with 4-fluorotoluene as an internal standard

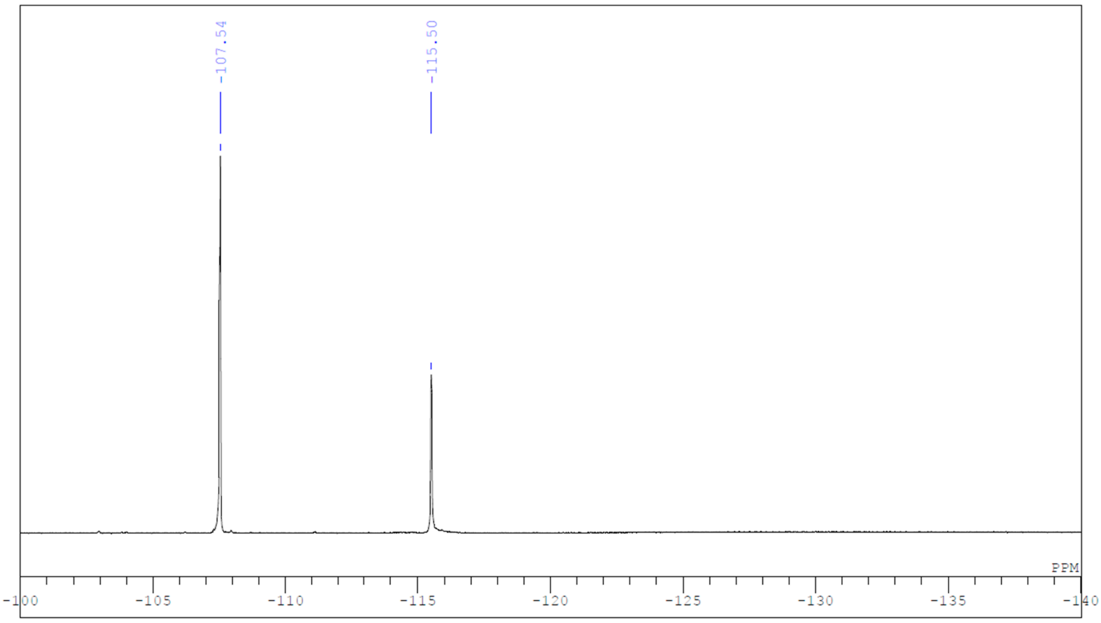

Methyl 7-bromo-3-methyl-1-oxo-1H-isochromene-4-carboxylate (3ga)

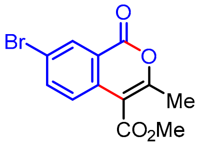

^{1}H NMR (400 MHz, $CDCl_3$)

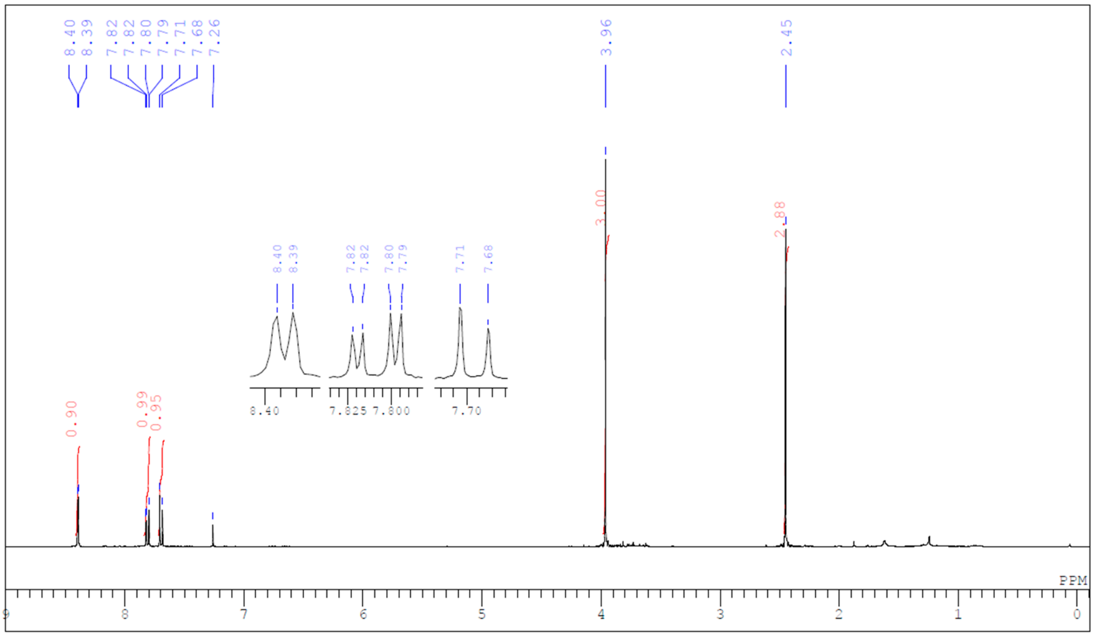

$^{13}C\{^{1}H\}$ NMR (101 MHz, $CDCl_3$)

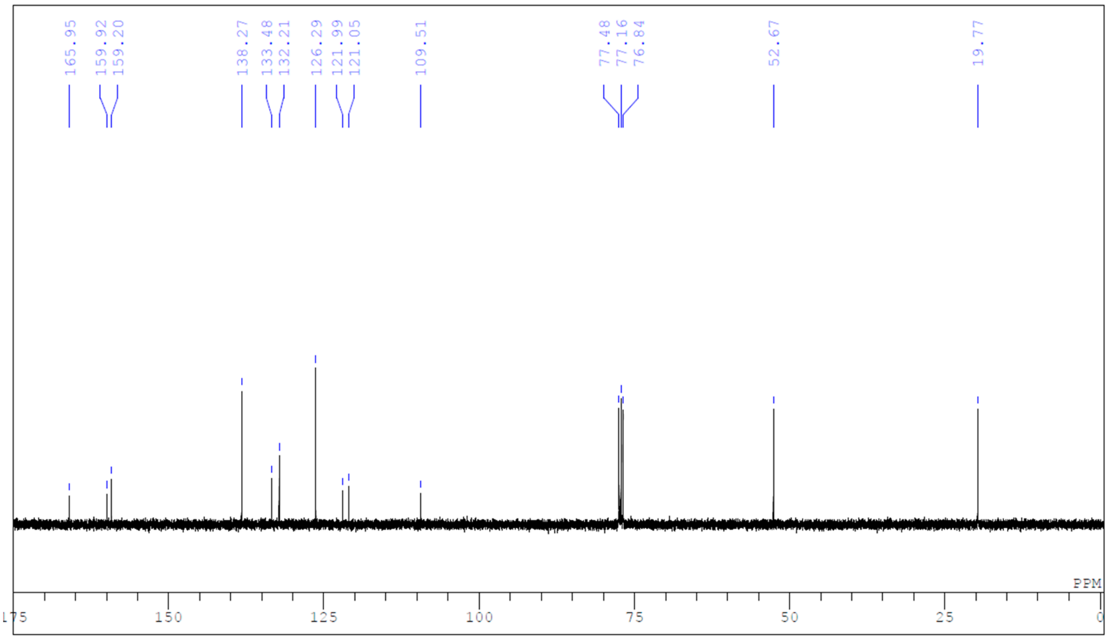

Methyl 6-chloro-3-methyl-1-oxo-1H-isochromene-4-carboxylate (3ha)

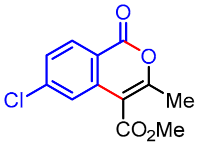

^{1}H NMR (400 MHz, $CDCl_3$)

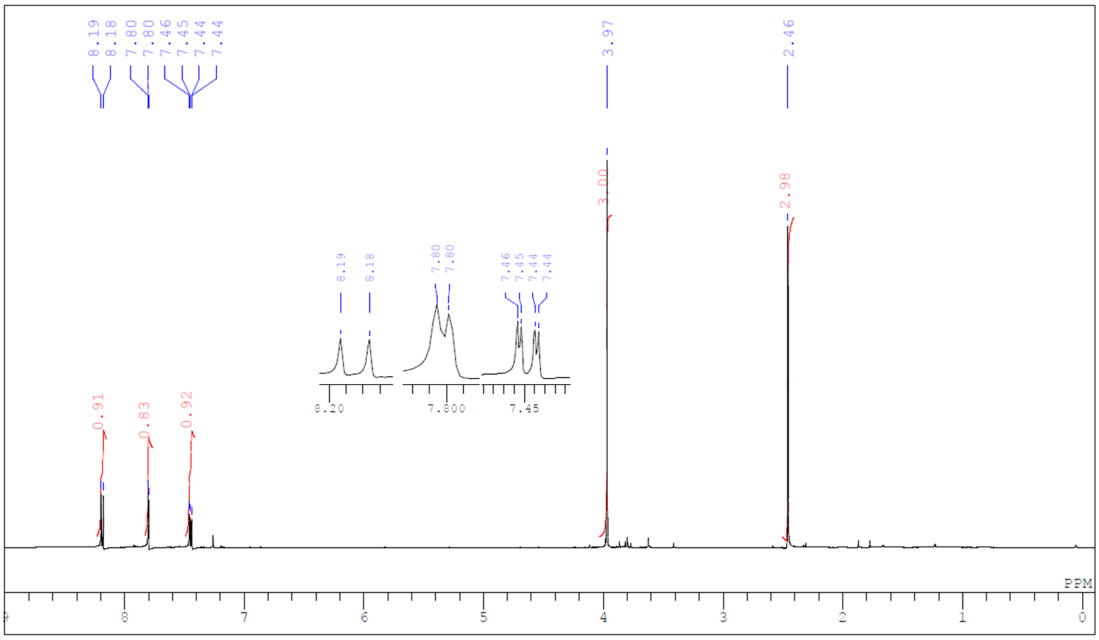

$^{13}C\{^{1}H\}$ NMR (101 MHz, $CDCl_3$)

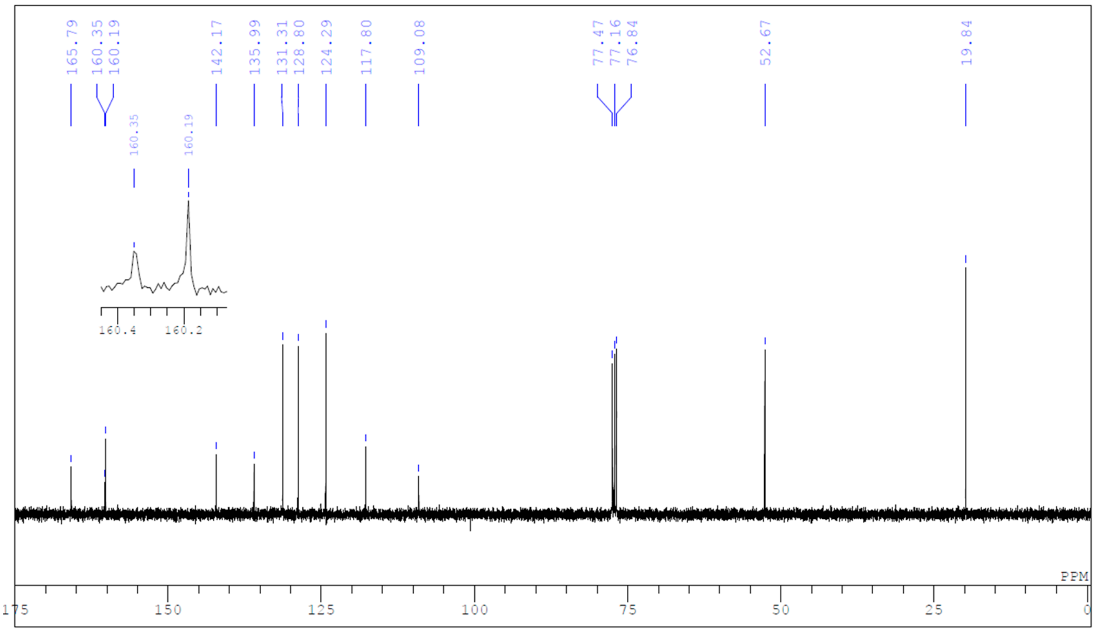

Methyl 3,7-dimethyl-1-oxo-1H-isochromene-4-carboxylate (3ia)

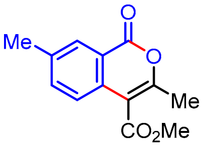

^1H NMR (400 MHz, $CDCl_3$)

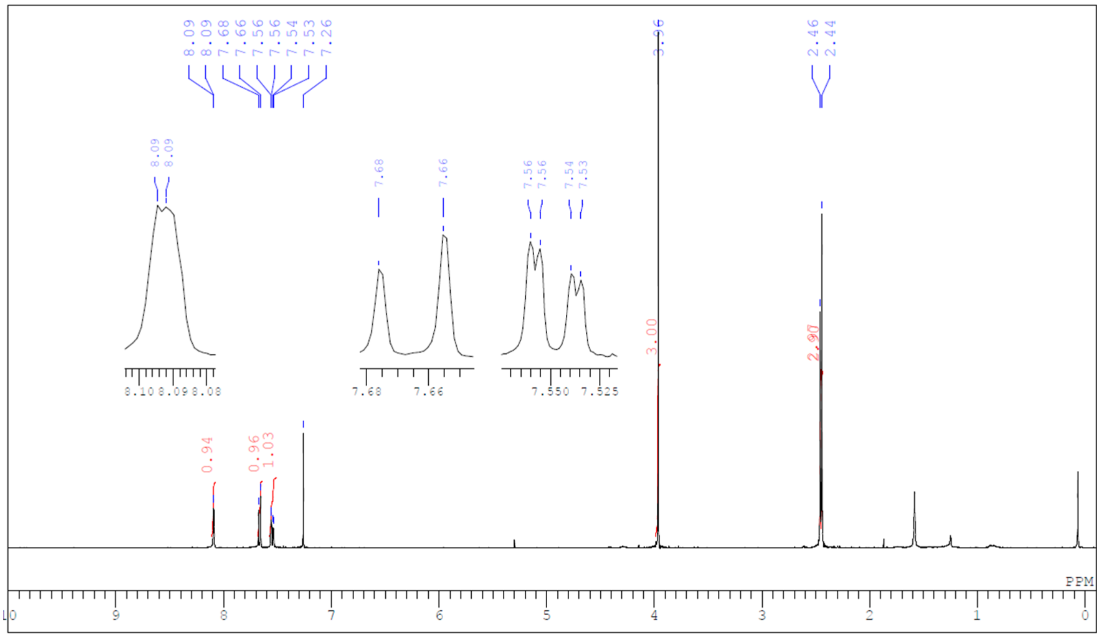

$^{13}C\{^1H\}$ NMR (101 MHz, $CDCl_3$)

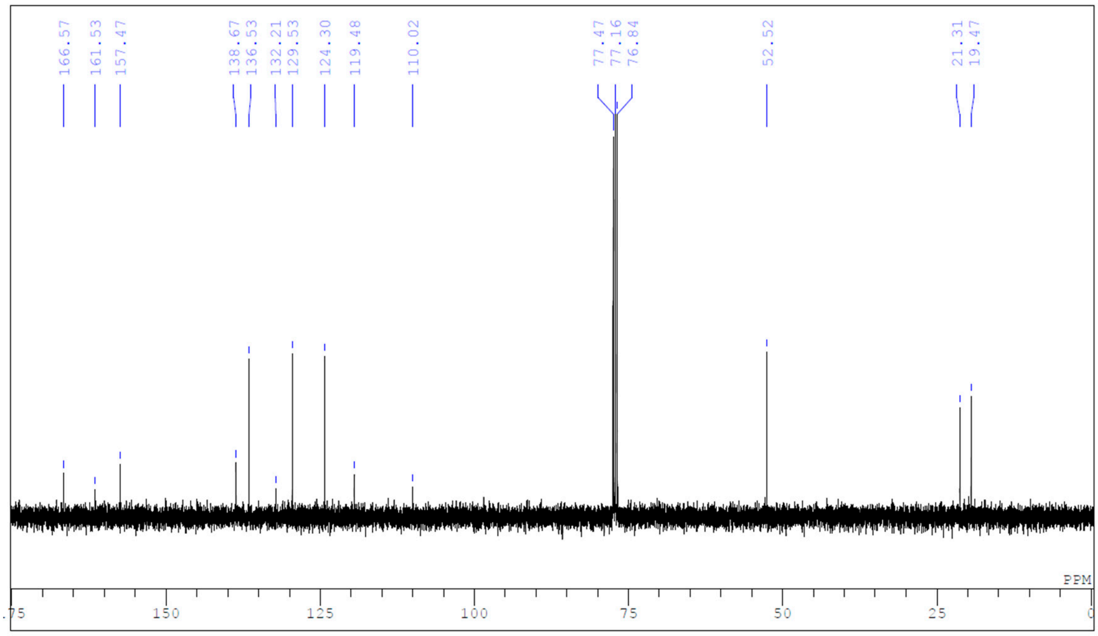

Methyl 3-methyl-1-oxo-1H-benzo[g]isochromene-4-carboxylate (3ja)

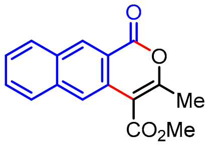

^{1}H NMR (400 MHz, CDCl$_3$)

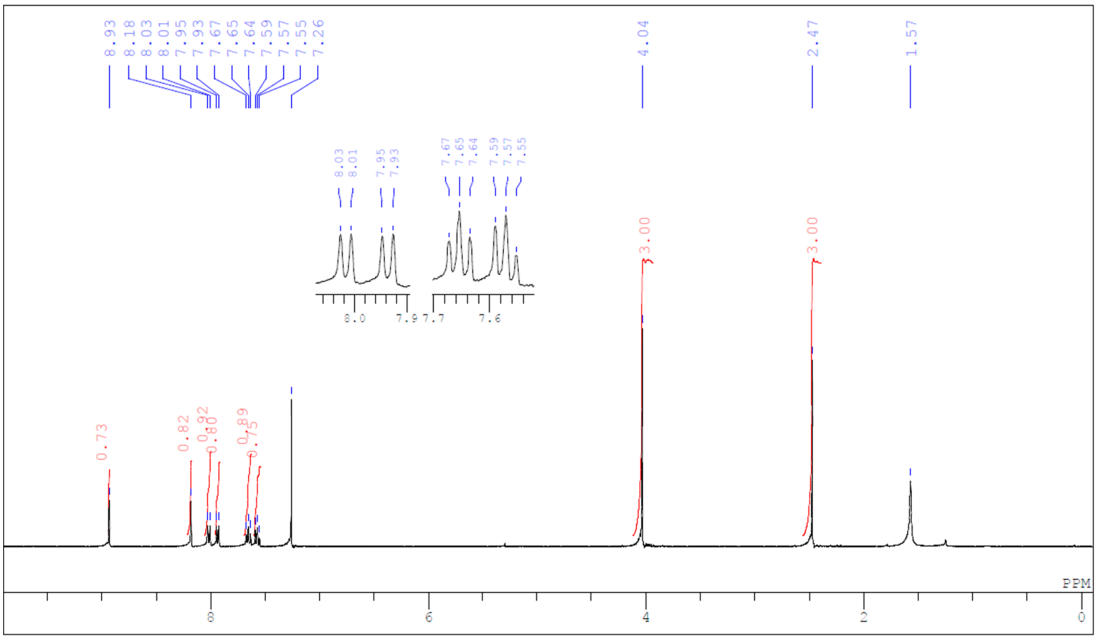

^{13}C{^{1}H} NMR (101 MHz, CDCl$_3$)

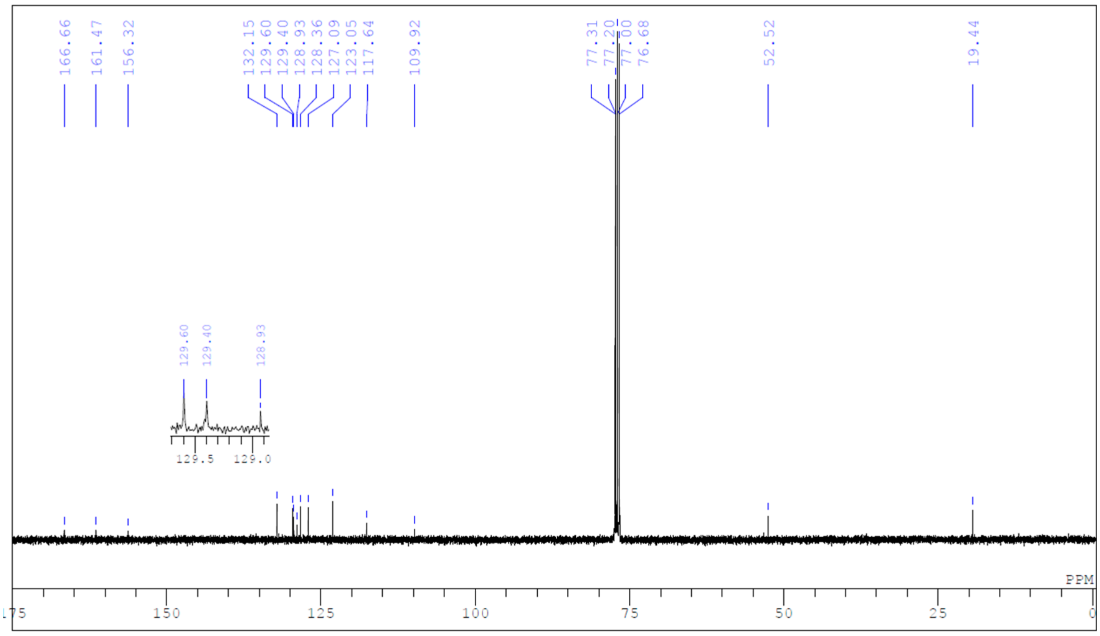

Methyl 5-methyl-7-oxo-7H-thieno[2,3-c]pyran-4-carboxylate (3ka)

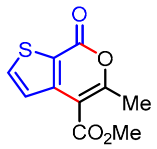

^{1}H NMR (400 MHz, $CDCl_3$)

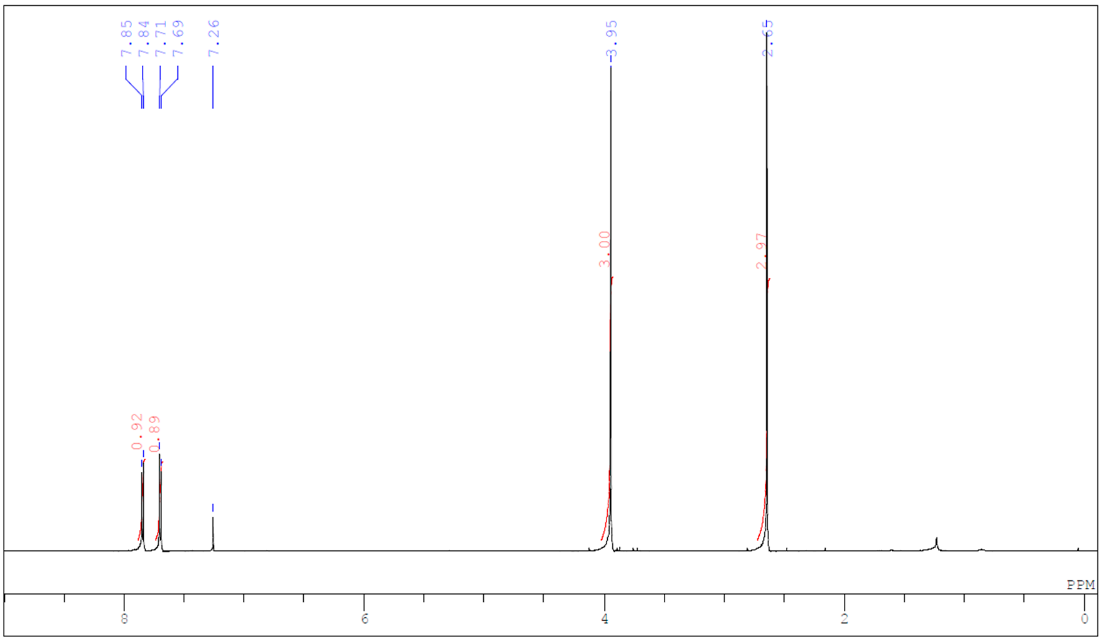

$^{13}C\{^1H\}$ NMR (101 MHz, $CDCl_3$)

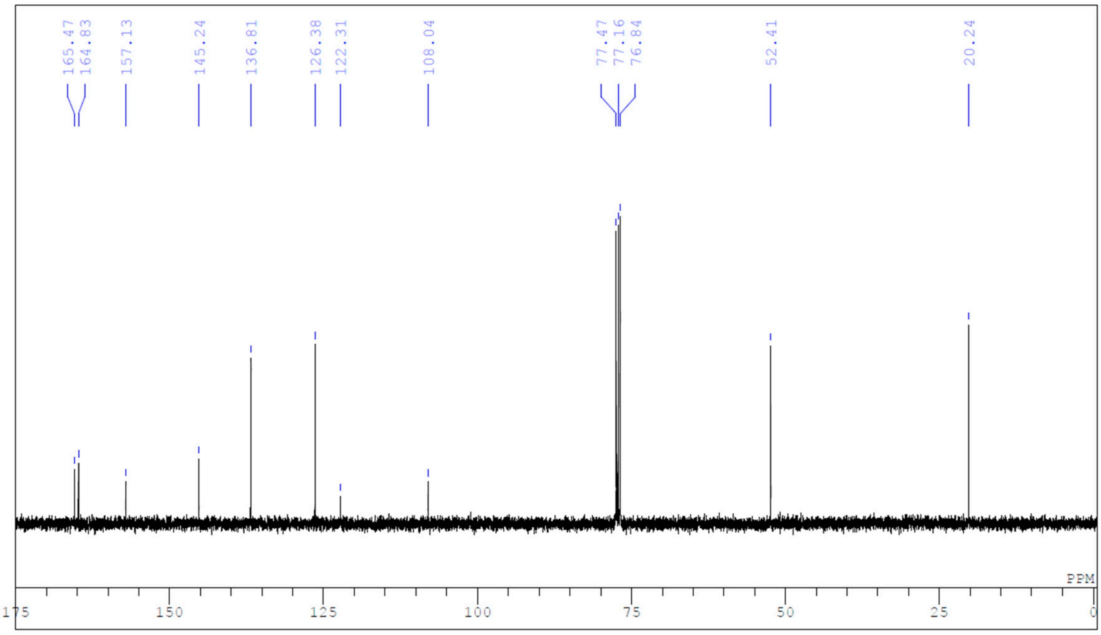

Methyl 1-imino-3-methyl-1H-isochromene-4-carboxylate (3la)

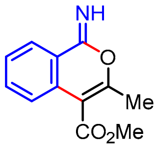

^{1}H NMR (400 MHz, $CDCl_3$)

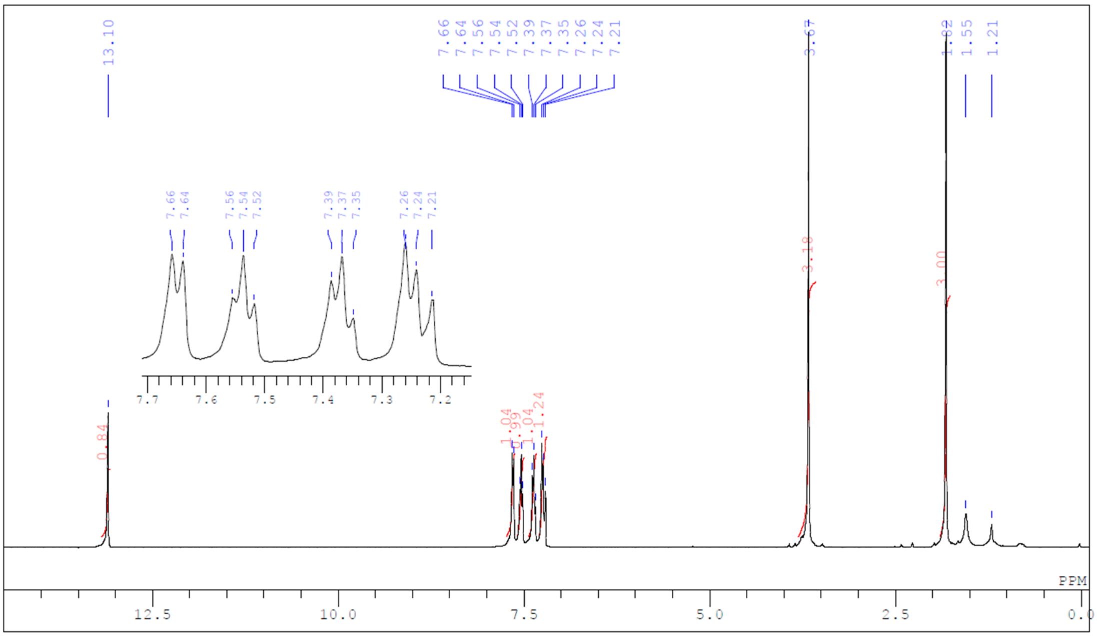

^{13}C{^{1}H} NMR (101 MHz, $CDCl_3$)

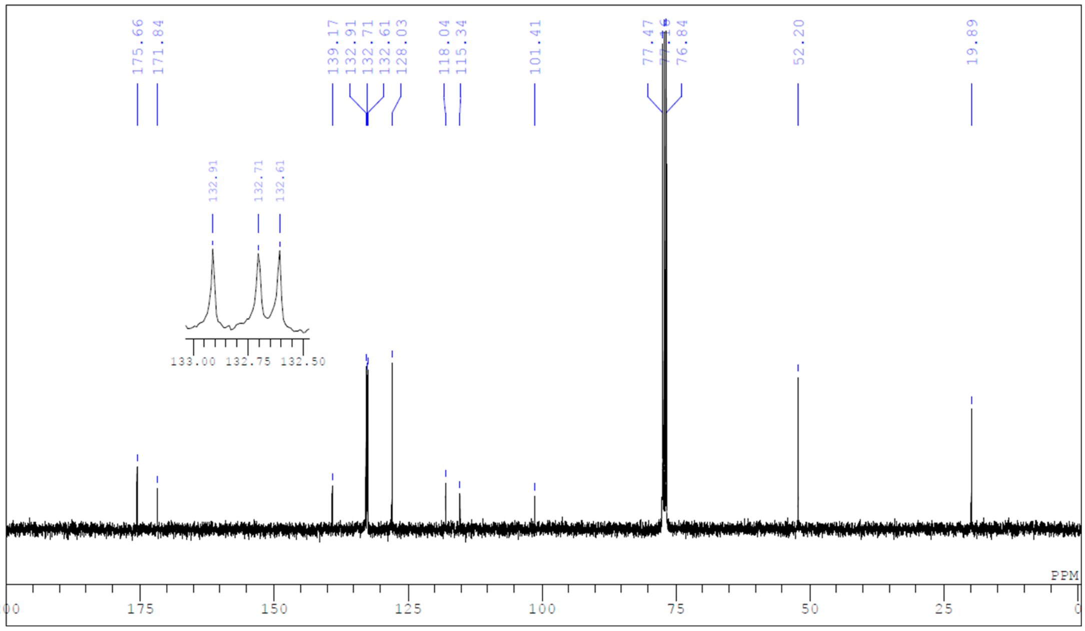

Ethyl 3-methyl-1-oxo-1H-isochromene-4-carboxylate (3ab)

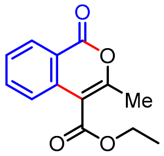

^{1}H NMR (400 MHz, $CDCl_3$)

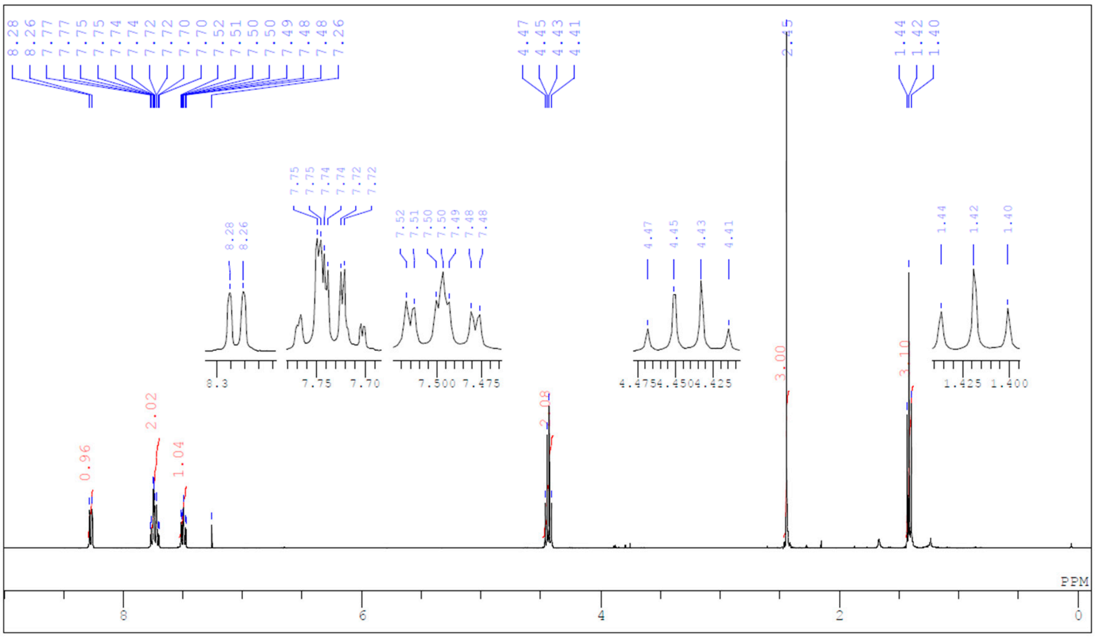

$^{13}C\{^{1}H\}$ NMR (101 MHz, $CDCl_3$)

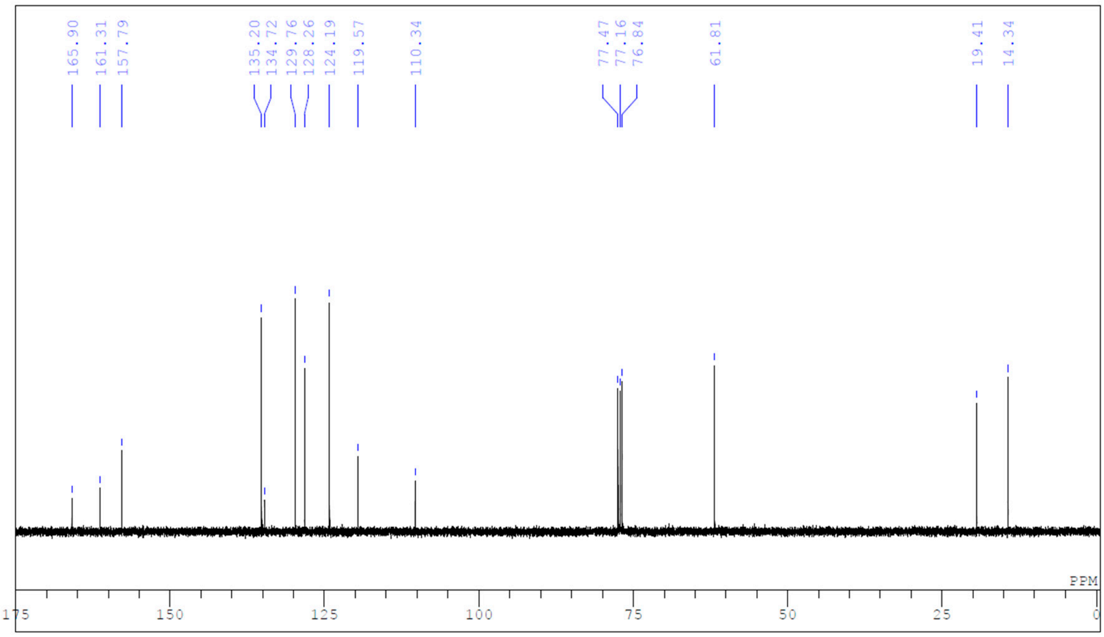

***tert*-Butyl 3-methyl-1-oxo-1H-isochromene-4-carboxylate (3ac)**

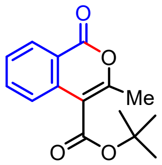

^{1}H NMR (400 MHz, $CDCl_3$)

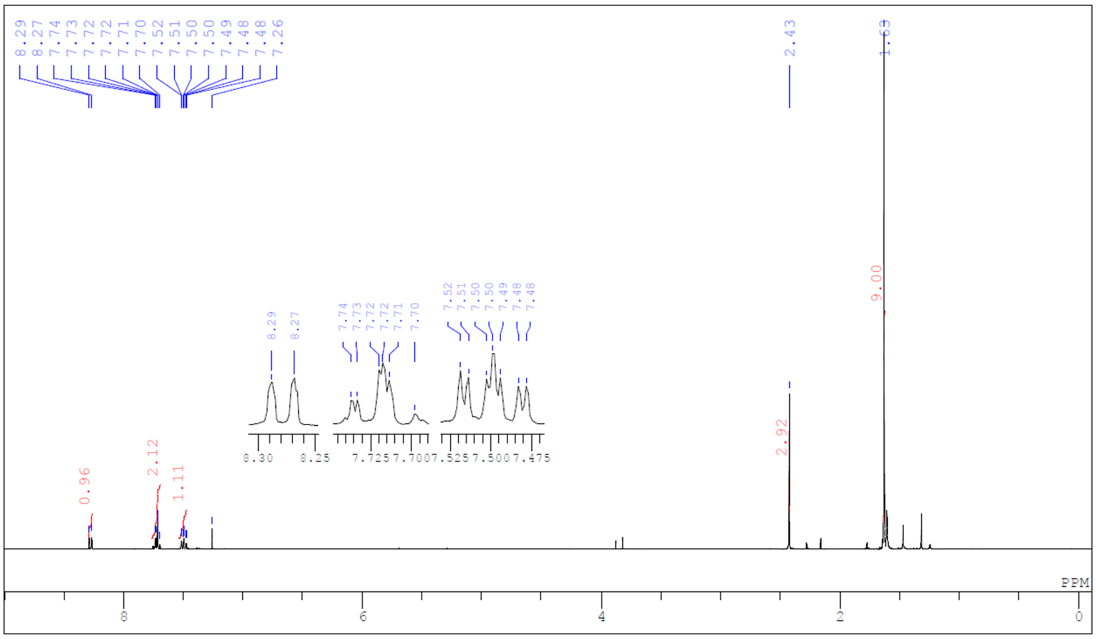

^{13}C{^{1}H} NMR (101 MHz, $CDCl_3$)

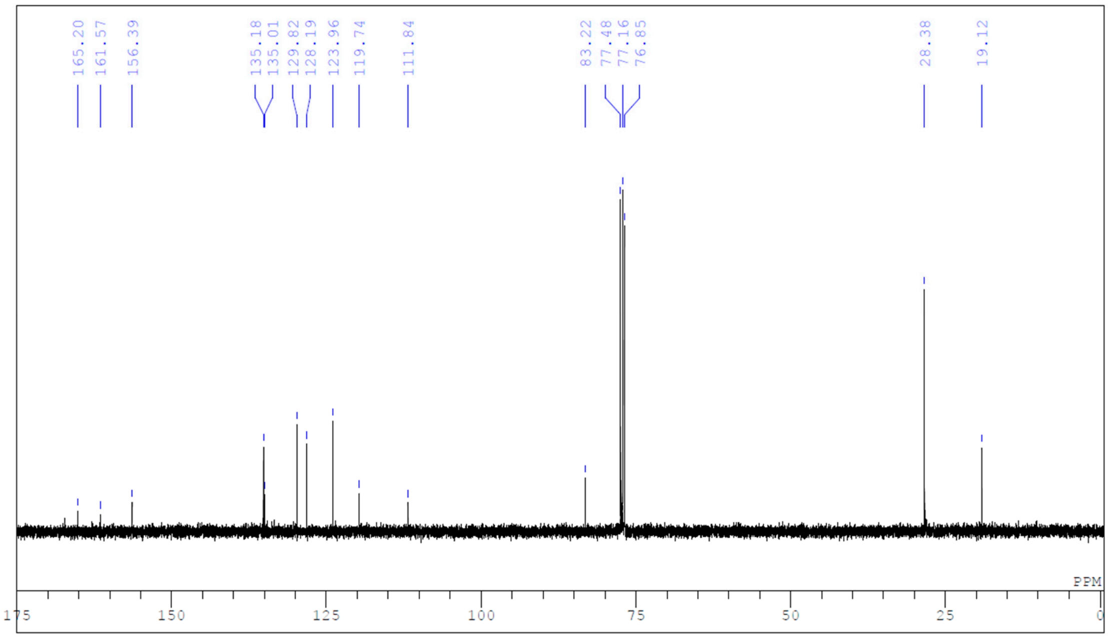

Allyl 3-methyl-1-oxo-1H-isochromene-4-carboxylate (3ad)

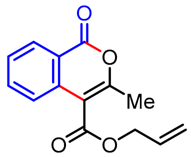

^{1}H NMR (400 MHz, CDCl$_3$)

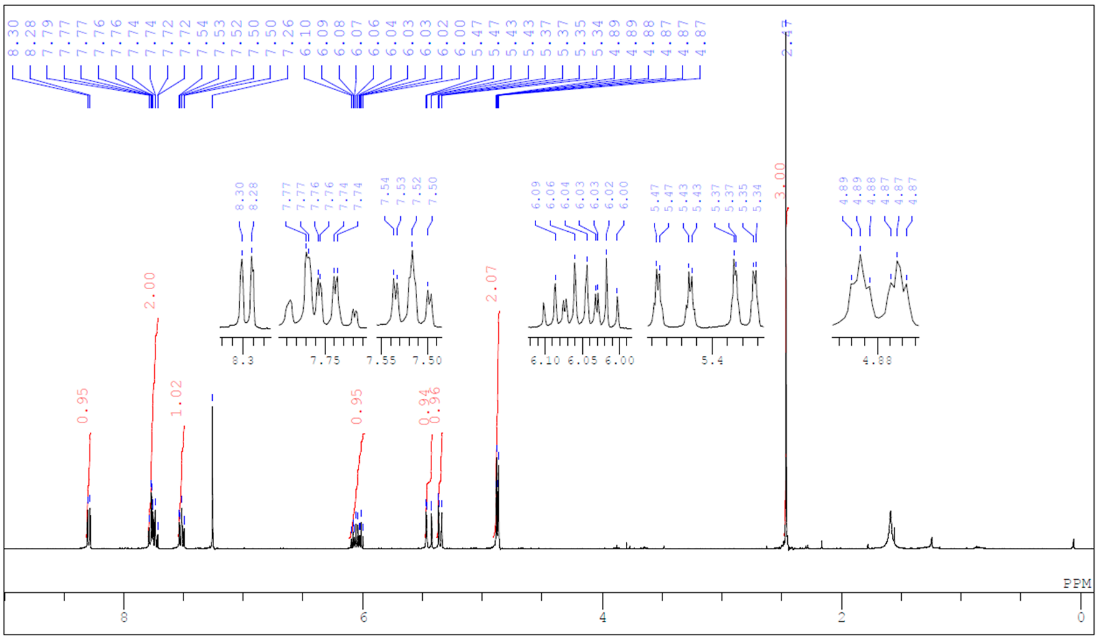

^{13}C{^{1}H} NMR (101 MHz, CDCl$_3$)

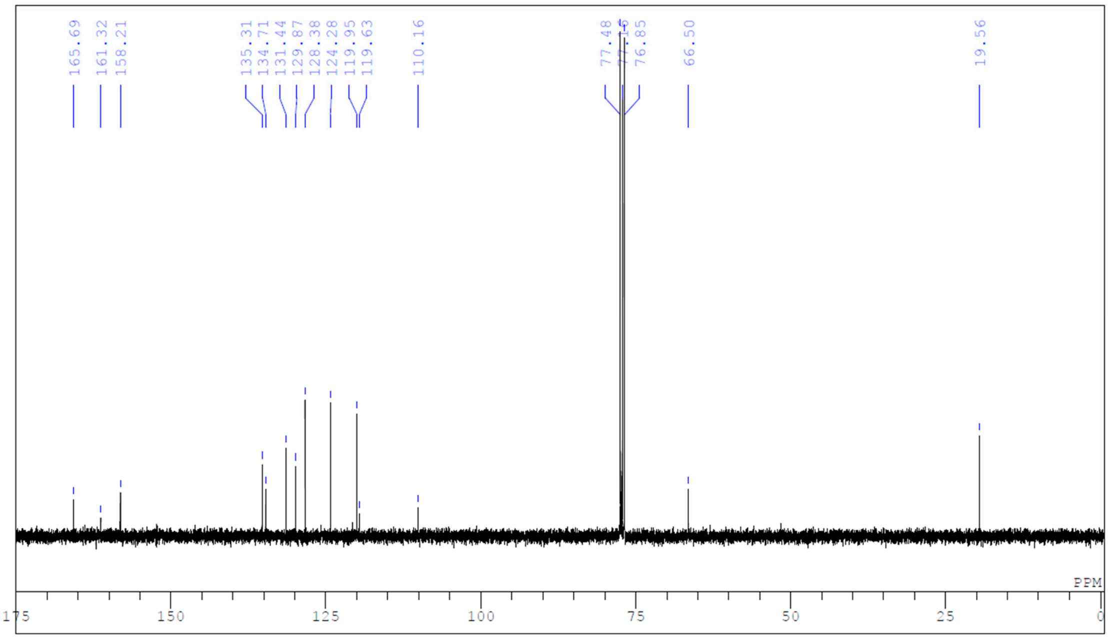

Methyl 3-ethyl-1-oxo-1H-isochromene-4-carboxylate (3ae)

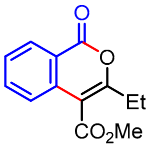

^{1}H NMR (400 MHz, $CDCl_3$)

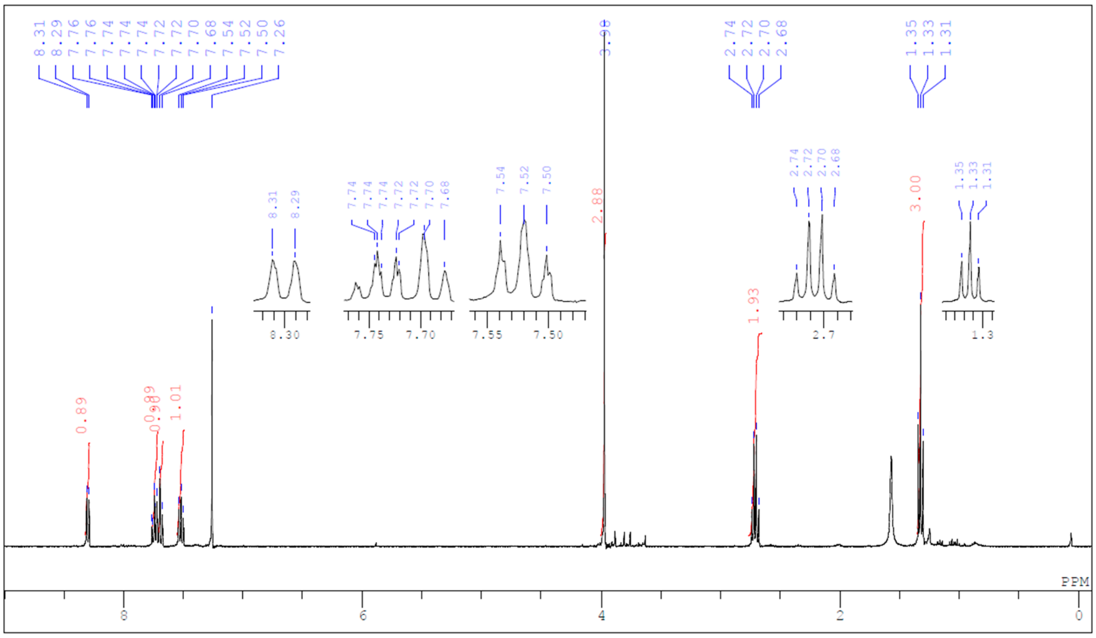

$^{13}C\{^{1}H\}$ NMR (101 MHz, $CDCl_3$)

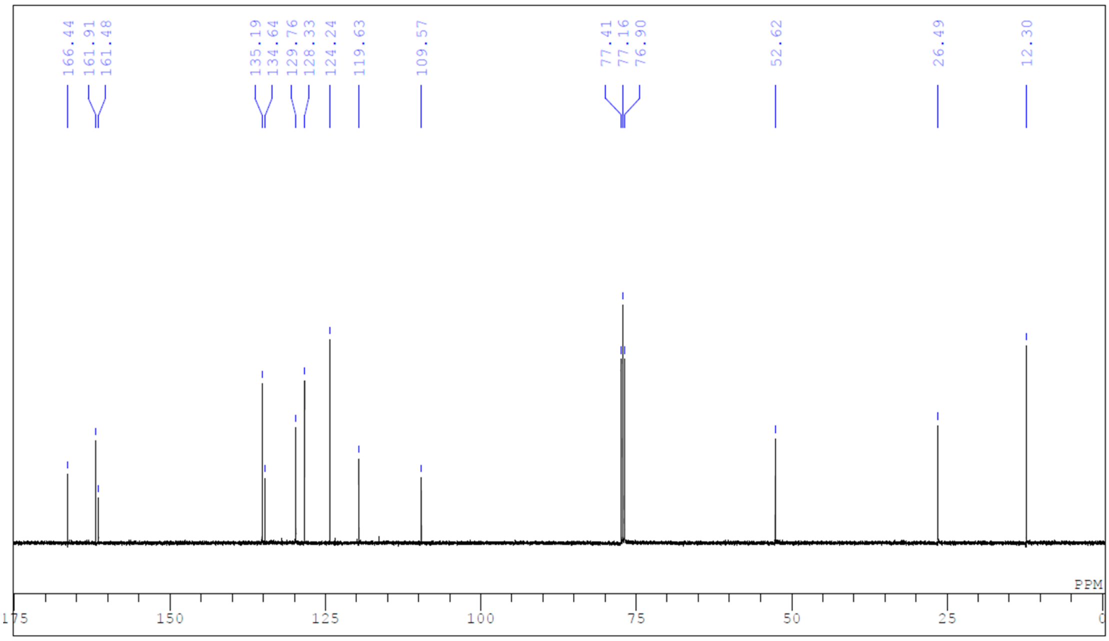

3-Methyl-1-oxo-1H-isochromene-4-carbonitrile (4)

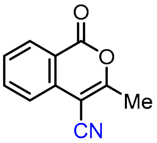

^{1}H NMR (400 MHz, CDCl$_3$)

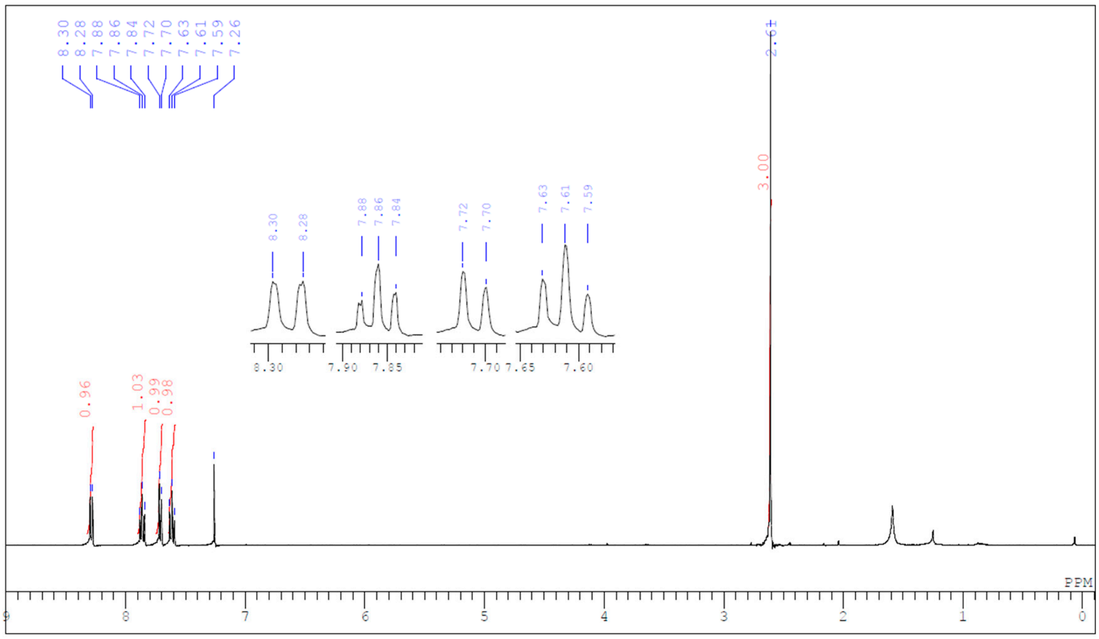

^{13}C{^{1}H} NMR (101 MHz, CDCl$_3$)

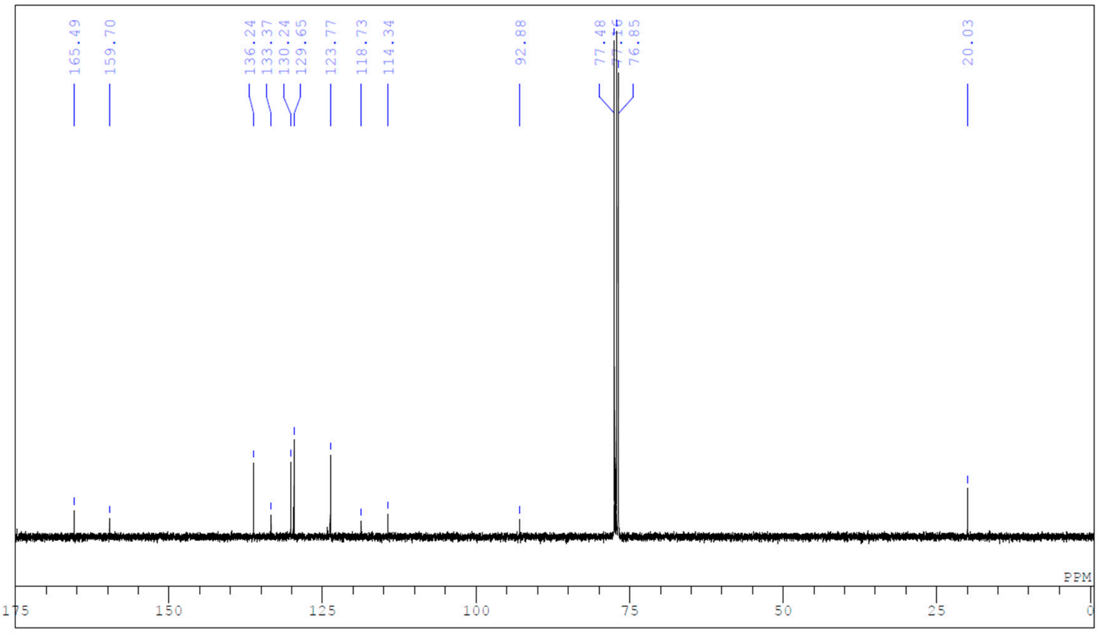

Methyl 3-methyl-1-oxo-1,2-dihydroisoquinoline-4-carboxylate (5)

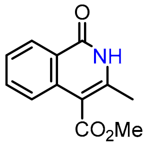

^{1}H NMR (400 MHz, DMSO-d$_6$)

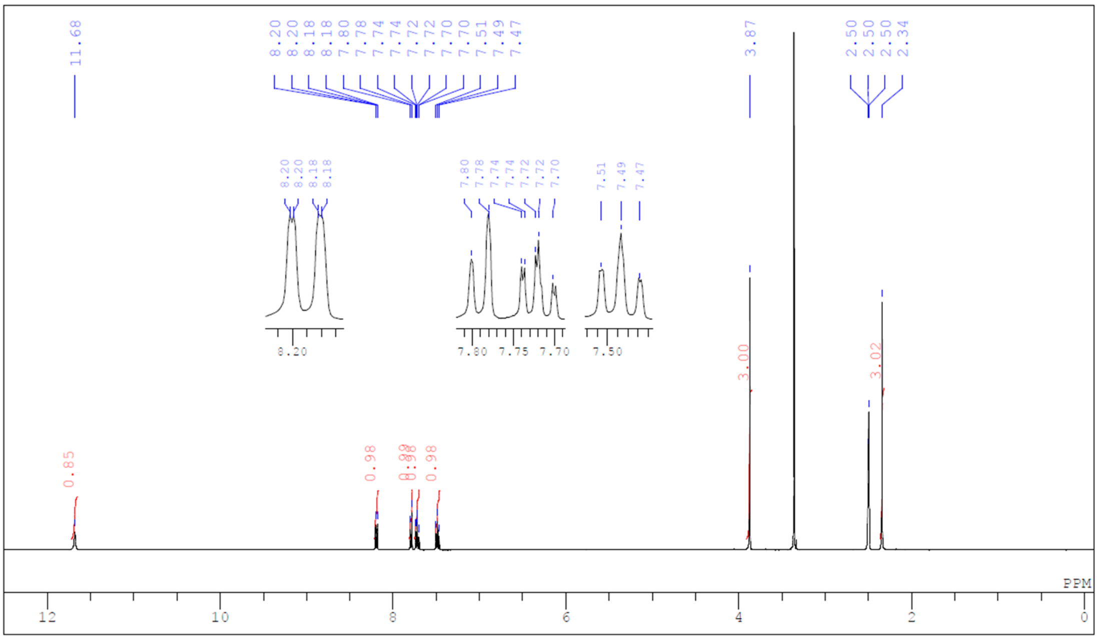

^{13}C{^{1}H} NMR (101 MHz, DMSO-d$_6$)

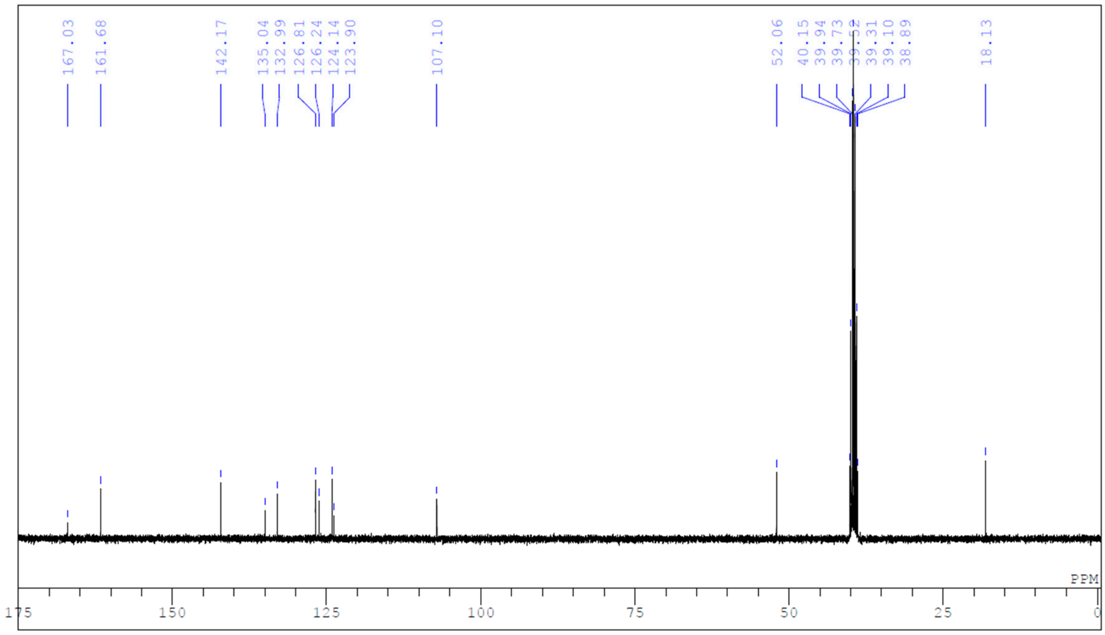

Methyl 3-(bromomethyl)-1-oxo-1H-isochromene-4-carboxylate (6)

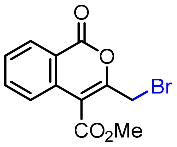

^1H NMR (400 MHz, $CDCl_3$)

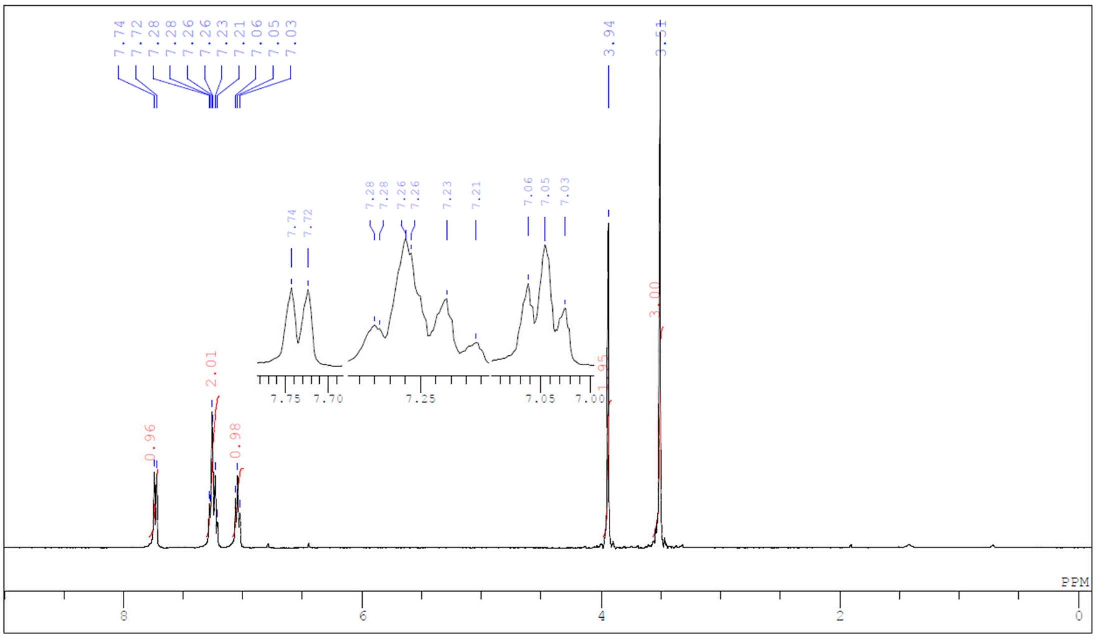

$^{13}C\{^1H\}$ NMR (101 MHz, $CDCl_3$)

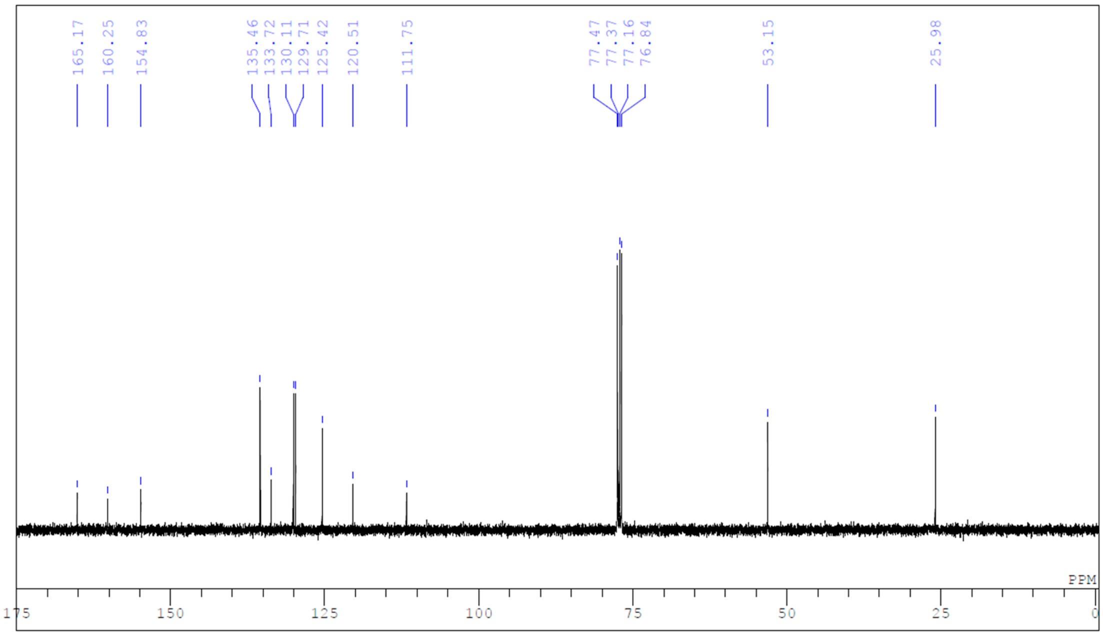

Methyl 1-oxo-3-(phenoxymethyl)-1H-isochromene-4-carboxylate (7)

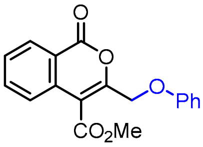

^{1}H NMR (400 MHz, $CDCl_3$)

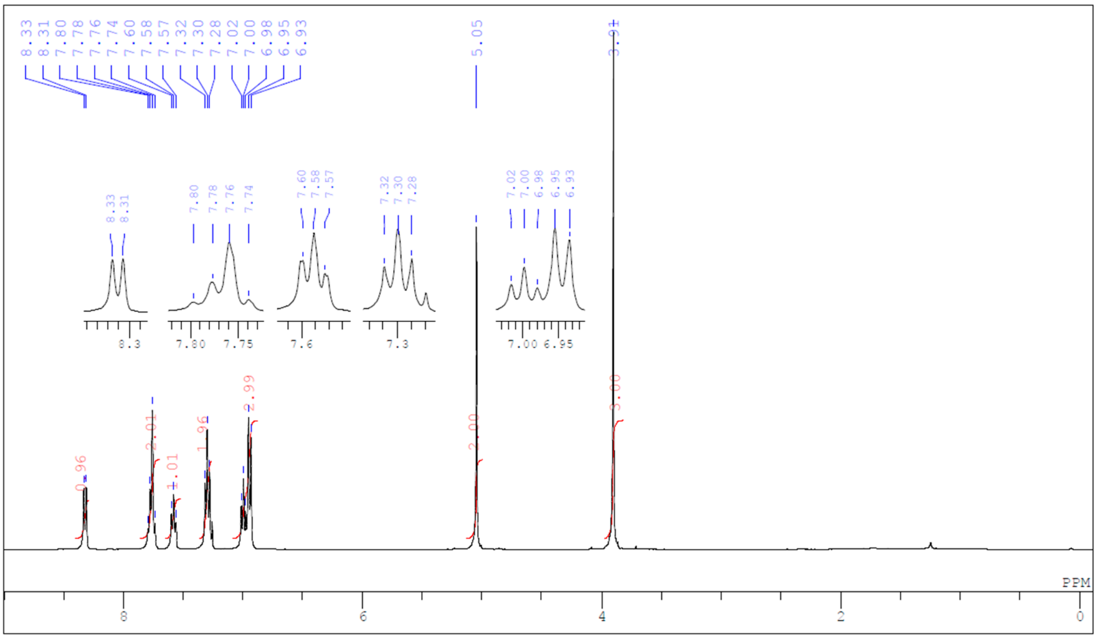

$^{13}C\{^1H\}$ NMR (101 MHz, $CDCl_3$)

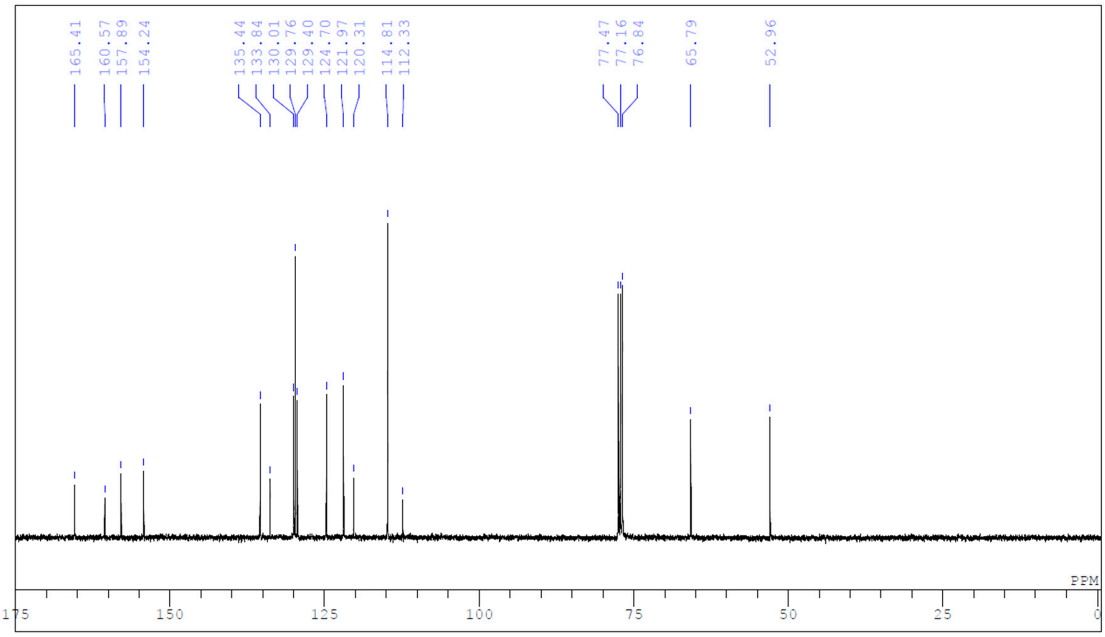

Methyl 3-(azidomethyl)-1-oxo-1H-isochromene-4-carboxylate (8)

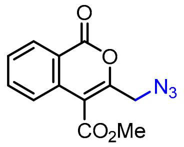

^{1}H NMR (400 MHz, $CDCl_3$)

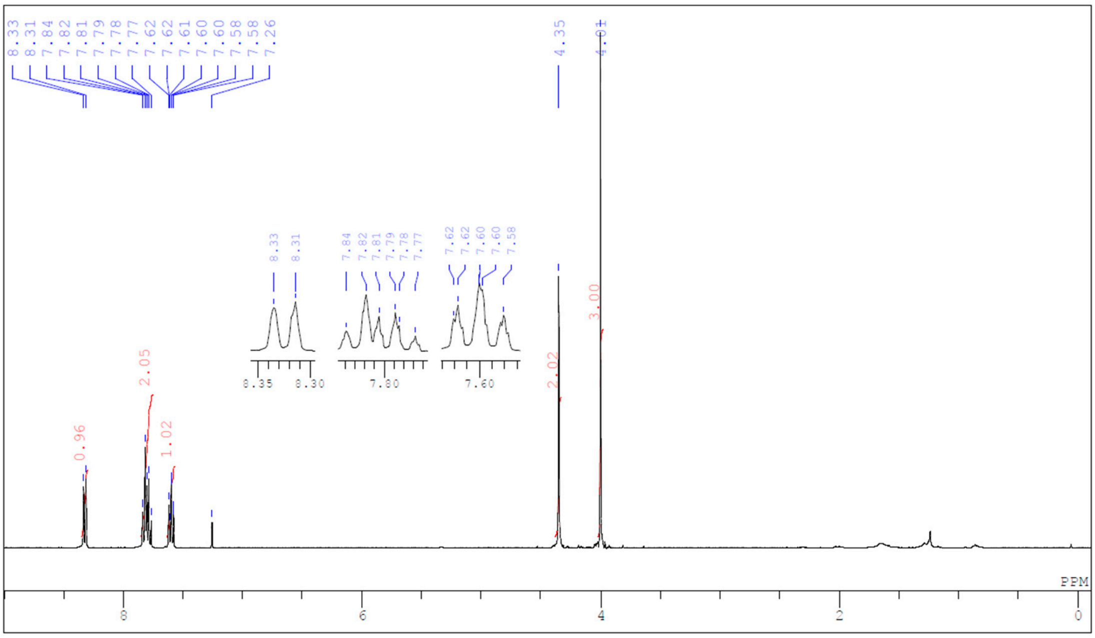

^{13}C{^{1}H} NMR (101 MHz, $CDCl_3$)

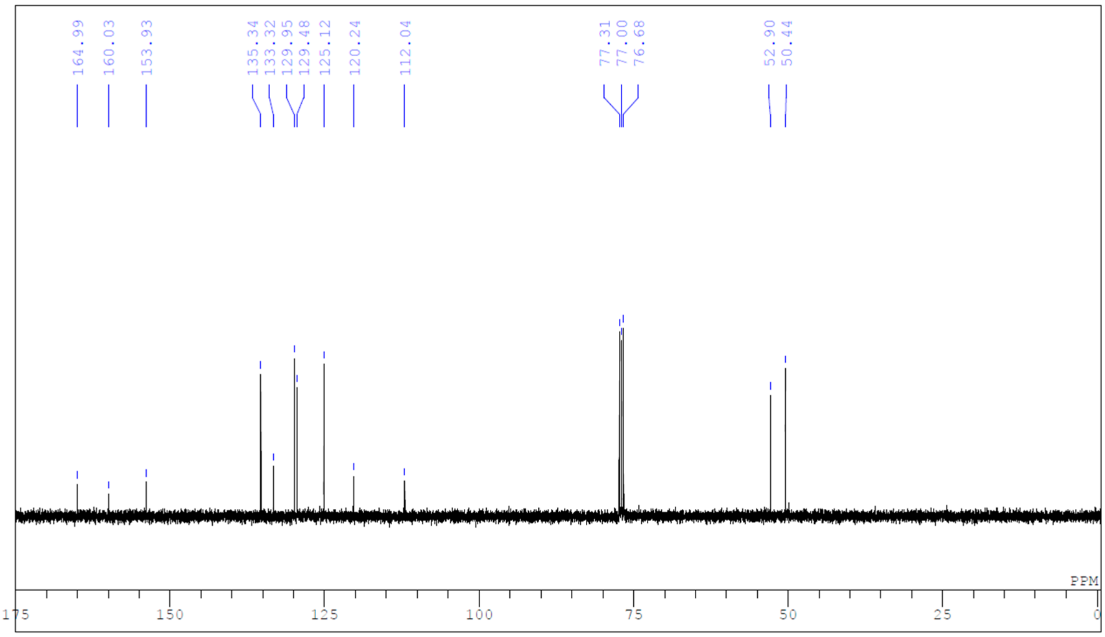

Methyl 3-((diethoxyphosphoryl)methyl)-1-oxo-1H-isochromene-4-carboxylate (9)

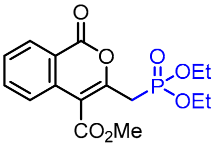

^{1}H NMR (400 MHz, $CDCl_3$)

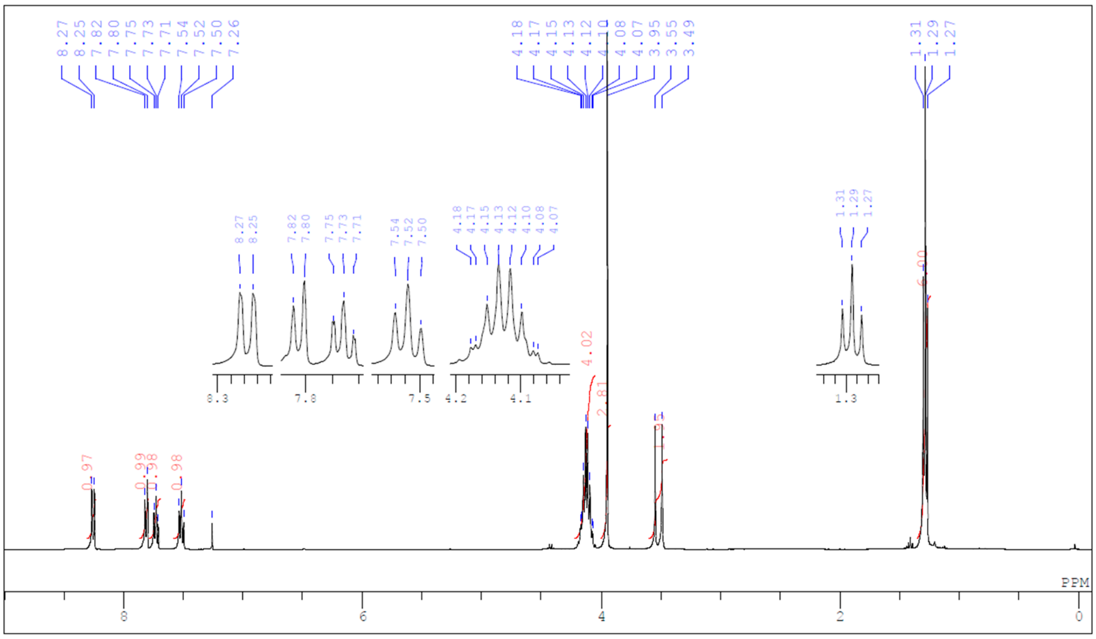

$^{13}C\{^{1}H\}$ NMR (101 MHz, $CDCl_3$)

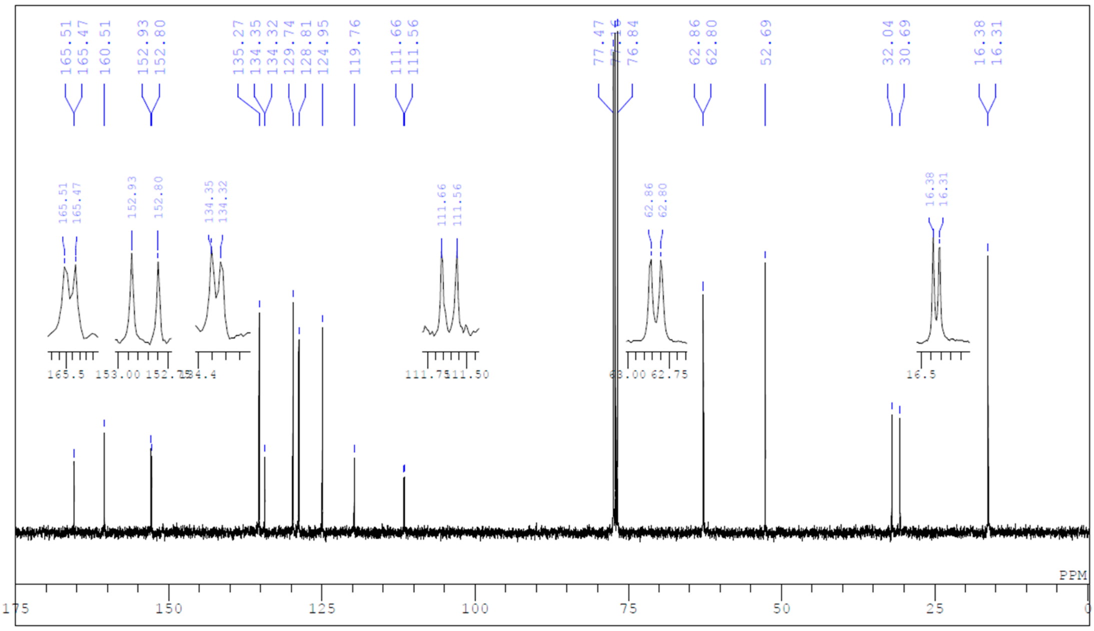

1H-Furo[3,4-c]isochromene-1,5(3H)-dione (10)

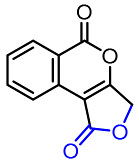

^{1}H NMR (400 MHz, $CDCl_3$)

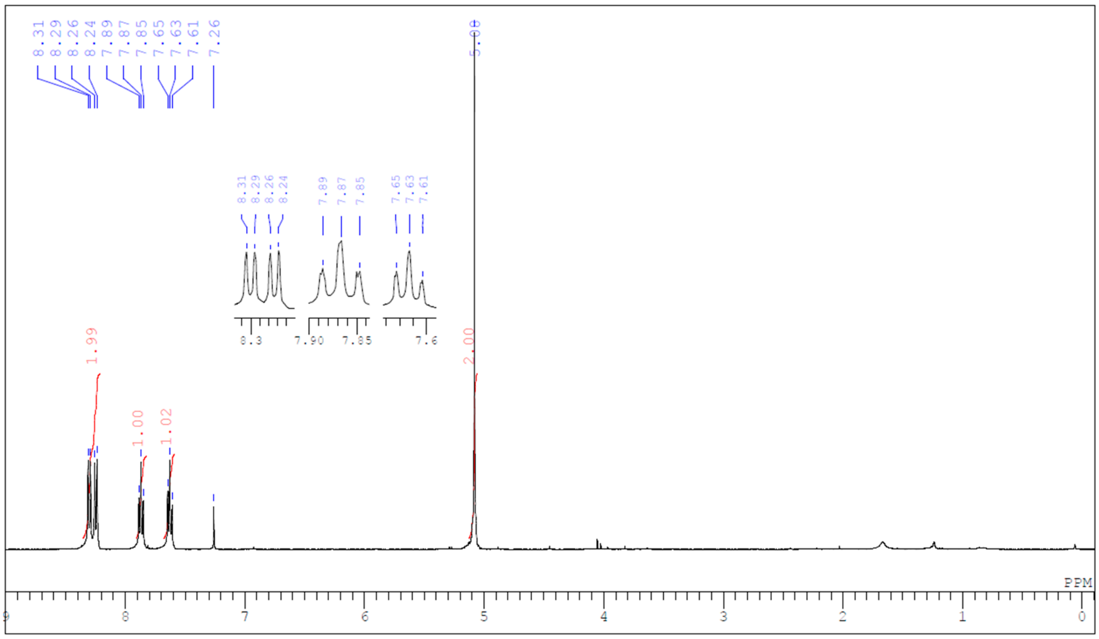

$^{13}C\{^{1}H\}$ NMR (101 MHz, $CDCl_3$)

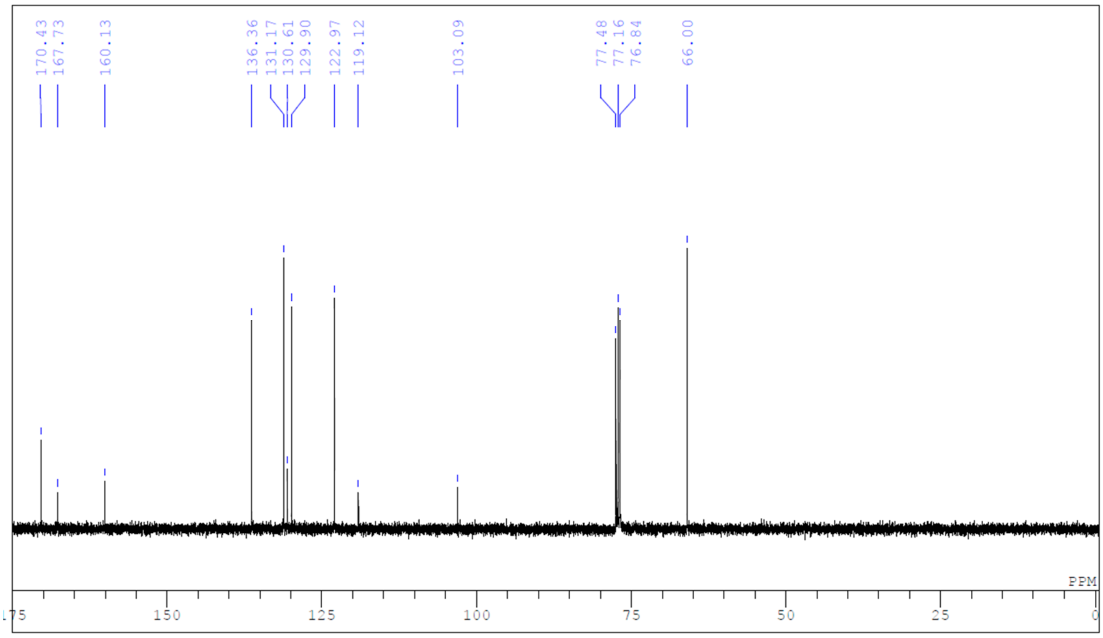

Supplement: Supplementary file 1 [file molecules-31-01069-s001.zip › molecules-4180711-supplementary.pdf]
